# Modulation of cardiac fatty acid or glucose oxidation to treat heart failure in preclinical models: a systematic review and meta-analysis

**DOI:** 10.1038/s43856-025-00924-5

**Published:** 2025-06-04

**Authors:** Tom Fischer, Christina Schenkl, Estelle Heyne, Peter Schlattmann, Torsten Doenst, P. Christian Schulze, T. Dung Nguyen

**Affiliations:** 1 Department of Medicine I (Cardiology, Angiology, Intensive Care Medicine), Jena University Hospital, Friedrich Schiller University Jena, Jena, Germany; 2 Department of Cardiothoracic Surgery, Jena University Hospital, Friedrich Schiller University Jena, Jena, Germany; 3 Institute of Medical Statistics, Computer and Data Sciences, Jena University Hospital, Friedrich Schiller University Jena, Jena, Germany

**Keywords:** Heart failure, Energy metabolism

## Abstract

**Background:**

Current expert opinion on cardiac metabolism in heart failure (HF) suggests that inhibiting cardiac fatty acid oxidation (FAO) or stimulating cardiac glucose oxidation (GO) can improve heart function. However, systematic evidence is lacking, and contradictory data exist. Therefore, we conducted a comprehensive meta-analysis to assess the effects of modulating myocardial GO or FAO on heart function.

**Methods:**

We screened MEDLINE via Ovid, Scopus, and Web of Science until March 02, 2024 for interventional studies reporting significant changes in cardiac GO or FAO in established animal models of HF, such as ischemia-reperfusion, pressure overload, rapid pacing, and diabetic cardiomyopathy. We employed multivariate analysis (four-level random-effects model) to enclose all measures of heart function. Additionally, we used meta-regression to explore heterogeneity and contour-enhanced funnel plots to assess publication bias. The protocol is registered on PROSPERO (CRD42023456359).

**Results:**

Of a total of 10,628 studies screened, 103 studies are included. Multivariate meta-analysis reveals that enhancing cardiac GO considerably restores cardiac function (Hedges’ *g* = 1.03; 95% CI: 0.79–1.26; *p* < 0.001). Interestingly, interventions associated with reduced myocardial FAO show neutral effects (Hedges’ *g* = 0.24; 95% CI: −0.57–1.05; *p* = 0.557), while those augmenting myocardial FAO markedly improve function (Hedges’ *g* = 1.17; 95% CI: 0.58–1.76; *p* < 0.001).

**Conclusions:**

Our data underscore the role of cardiac metabolism in treating HF. Specifically, these results suggest that stimulating either myocardial FAO or GO may considerably improve cardiac function. Furthermore, these results question the current notion that inhibition of cardiac FAO is protective.

## Plain language summary

The heart mainly derives its energy by burning fat and sugar in a process called oxidation. In heart failure, a condition where the heart cannot pump blood effectively, fat and sugar oxidation of the heart are substantially affected. Targeting energy metabolism may therefore carry therapeutic potential. However, existing data are conflicting on how to best modulate these processes. By systematically evaluating published studies using animal models, we found that treatments associated with increased oxidation of fats or sugar significantly improve heart function. These results provide a foundation for the development of new heart failure drugs and challenge the prevailing notion that limiting fat oxidation is beneficial in heart failure.

## Abstract (web summary)

Fischer et al. conduct a comprehensive meta-analysis to investigate the effects of modulating myocardial metabolism in heart failure. Contrary to the common notion, they discover that stimulating either myocardial fatty acid oxidation or glucose oxidation may considerably improve cardiac function.

## Introduction

Despite significant advances in drug and device therapy, the mortality of heart failure (HF) remains high^1^. Especially in the context of heart failure with preserved ejection fraction (HFpEF), treatment options are still limited^2^.

The healthy heart uses fatty acid and glucose as its primary substrates^3^. During the progression to HF, several changes in cardiac fatty acid and glucose metabolism have been identified as parts of the heart’s metabolic remodelling^4,5^. Modulating pathways of fatty acid and glucose metabolism has been shown to affect cardiac function in animal models of HF^6,7^.

Initial studies in certain models of cardiac dysfunction have suggested inhibition of myocardial fatty acid oxidation (FAO) to improve oxygen efficiency leading to the development of several metabolic modulators such as trimetazidine, etomoxir, and perhexiline^8–12^. Interestingly, although the efficacy of some fatty acid inhibitors has been demonstrated in the context of HF, their protective effects are rarely associated with an actual suppression of cardiac FAO^13–15^. In addition, more recent data do not support the concept of inhibiting fatty acid utilisation to improve cardiac function^16–20^. For example, a human study even demonstrated that reducing myocardial fatty acid uptake by acipimox depressed cardiac power^21^. These inconsistencies remain a matter of debate, and some authors have suggested that the different etiologies of HF may play a role^8,22,23^.

Current expert opinion recommends inhibition of FAO or stimulation of glucose oxidation (GO) to treat HF^8,24^. There has been, however, no systematic evidence supporting these concepts. Our work aims to quantify the effects of modulating cardiac FAO and GO in established animal models of cardiac dysfunction to determine potential metabolic strategies to treat HF. Here, we show that stimulation of myocardial FAO or GO significantly improves cardiac function. Our findings challenge the prevailing concept that inhibiting cardiac FAO is protective and rather suggest augmenting myocardial substrate utilisation in general to treat HF.

## Methods

Our methodological approach was constructed in accordance with the guidelines set by Preferred Reporting Items for Systematic Reviews and Meta-Analyses (PRISMA) and was published in detail via the online platform PROSPERO on September 13, 2023 (CRD42023456359)^25^. An ethical approval is not applicable because this study is based exclusively on published literature and did not involve the collection of any new data requiring ethics review.

We performed a systematic literature search on Medline via Ovid, Web of Science, and Scopus from inception to March 2, 2024. Our search query was formulated to find all relevant animal studies in the domains of (1) heart failure; (2) glucose oxidation; and (3) fatty acid oxidation (see Supplementary Methods).

Papers were screened based on title and abstract, followed by full-text screening. Both processes were conducted by three independent reviewers (T.F., E.H., C.S.). In case of no consensus, a fourth person (T.D.N.) was consulted. As specified in the research protocol, we included established models of HF, such as ischemia-reperfusion (IR), pressure overload (PO), myocardial infarction (MI), rapid pacing, and diabetic cardiomyopathy (DMC). Combined models were excluded.

We decided to exclude data from human studies, because a reliable method for the direct measurement of cardiac substrate oxidation rates in humans is currently not available. Furthermore, papers were omitted if no significant reduction in at least one parameter of cardiac function was present in the untreated lesioned group. To be eligible, studies needed to perform interventions associated with significant changes in the rate of cardiac GO or FAO in lesioned animals, regardless of the type of intervention (e.g., drugs, genetic modifications, and dietary changes). Direct measurements of myocardial FAO and GO in vivo or ex vivo (e.g. using ^14^C or ^13^C marked substrates) were required. Combined interventions were excluded.

Three authors (T.F., E.H., C.S.) independently extracted data, and any discrepancies were reviewed with a fourth evaluator (T.D.N.). The following details were recorded: authors; year of publication; journal; animal information (species, sex, disease model, number of animals); type of intervention; changes in cardiac GO or FAO in consequence of the intervention; outcome measures (parameter, mean, SD, number of animals per group). The extracted study characteristics are in Supplementary Data 1 and the numerical outcome measures are in Supplementary Data 3.

We integrated 15 systolic, diastolic, morphological, and histological parameters to obtain a comprehensive assessment of cardiac function. Systolic parameters include rate pressure product, ejection fraction, fractional shortening, cardiac output, cardiac power, cardiac work, maximal rate of pressure rise, and left ventricular developed pressure. Diastolic parameters involve maximal rate of pressure fall, left ventricular end diastolic pressure, ratio of E-wave to e’-wave, and ratio of E-wave to A-wave. Morphological and histological measures encompass left ventricular end diastolic diameter, infarct size, and cardiac fibrosis.

The data was extracted from text and tables. We used WebPlotDigitizer to obtain data from figures^26^. Records with multiple intervention groups were treated as individual studies during the inclusion process and incorporated into the meta-analysis. If the same control group was used, these were adjusted to resemble the true effective number of controls per group^27^. We addressed missing data by contacting the corresponding author. If relevant data were not available or insufficient for quantitative analysis, studies were excluded.

Study quality was assessed using the CAMARADES checklist as follows: (1) peer reviewed publication; (2) control of temperature; (3) allocation concealment; (4) random allocation to treatment or control; (5) blinded assessment of outcome; (6) sample size calculation; (7) compliance with animal welfare regulations; and (8) statement of potential conflict of interests^28^. Attrition and selection bias were appraised using the SYRCLE Risk of bias tool^29^.

### Statistics and reproducibility

As mentioned in the previous section, the included studies have employed various measures of cardiac function, for instance echocardiographical and ex vivo parameters. Furthermore, methodological differences may exist within a specific measurement (e.g. staining protocol to quantify infarct size and cardiac fibrosis). Hence, we used Hedges’ g to standardize the effect sizes, thereby permitting direct comparisons across different parameters^30^. Hedges’ g is particularly applicable to data from animal studies because it adjusts for small sample sizes^27^.

A multivariate meta-analysis model with four levels was used to address the dependant data structure^31^. Level 1: Variance due to sampling error, Level 2: Variance due to correlating cardiac functional parameters, Level 3: Variance due to multiple within study interventions (if more than one intervention was performed), Level 4: Between-study variance. A random-effects model was fitted to the data. This was decided a priori because we expected relevant between-study variability. The amount of heterogeneity in multi-level models (i.e., σ^2^) was estimated using the restricted maximum-likelihood estimator. In addition to the estimate of σ^2^, the Q-test for heterogeneity and the I^2^ statistics are reported. Since high heterogeneity is expected when analysing studies including all types of metabolic change (e.g., GO↑, FAO↓), we used meta-regression to investigate the effects of distinct metabolic alterations on cardiac function. The animal disease model was also a variable included for meta-regression, to explore sources of heterogeneity. As commonly accepted, we performed meta-regression and Egger’s regression if at least 10 studies per moderator variable were available^27,32^. For visual assessment of potential publication bias, we used contour-enhanced funnel plots^33^. Additionally, we examined funnel plot asymmetry via a sample size-based precision estimate (inverse of sample size), because classical Egger’s regression may not adequately control type 1 error rates when used with standardized mean differences^34–36^. For sensitivity analysis, we used a leave-one-out procedure to examine whether overall findings are robust to potentially influential studies. Tests and confidence intervals were computed using the Knapp and Hartung method^37^. Statistical analyses were performed using R version 4.4.1 with the additional metafor package^38,39^.

### Reporting summary

Further information on research design is available in the Nature Portfolio Reporting Summary linked to this article.

## Results

**Fig. 1:**
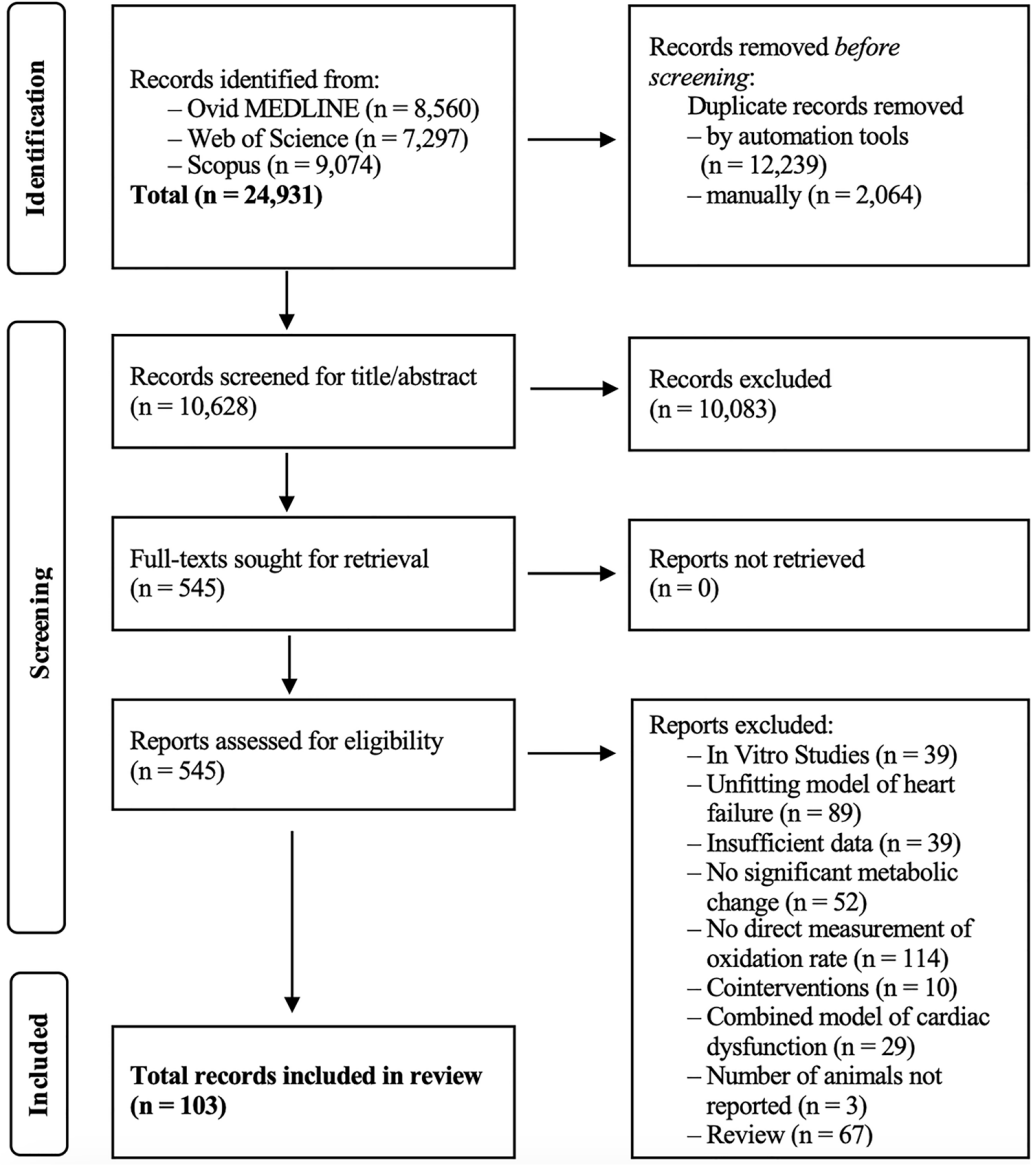
PRISMA flow diagram of systematic study selection. PRISMA flow chart summarising study selection process. Of a total of 10,628 studies screened, 103 studies are used for the meta-analysis.

In total, we identified 10,628 reports from our final search on March 2, 2024, after removing all duplicates. Subsequent title–abstract screening and full-text screening returned 103 studies eligible for meta-analysis. The reasons for exclusion in the full-text screening step are outlined in Fig. 1. Three papers were excluded due to missing sample sizes, which could not be resolved after contacting the corresponding authors. In several of the 103 papers, researchers conducted more than one intervention, leading to the inclusion of 120 studies in total into the meta-analysis. Animals (*n* = 2022) were predominantly male (~75%), rats (*n* = 952), mice (*n* = 851), and followed by rabbits (*n* = 92), dogs (*n* = 84) and swine (*n* = 43). Because significant differences between rodents and non-rodent species may exist, we also conducted separate analyses for both animal groups. Data obtained exclusively from rodents are presented in Supplementary Table 1. For non-rodent animals, analysis was not possible because of the small number of studies (*n* < 10). The interventions induced eight possible combinations of metabolic alterations. These are displayed in Table 1, along with the corresponding number of studies for each. The disease models used were IR (*n* = 70, 58%), PO (*n* = 27, 23%), DMC (*n* = 14, 12%), MI (*n* = 5, 4%), rapid pacing (*n* = 4, 3%). All included studies and their study characteristics are listed in Supplementary Data 1.

**Table 1:** Number of studies grouped by metabolic changes

Metabolic changes	Number of studies
GO↑	49
FAO↓GO↑	16
FAO↑	15
FAO↓	13
FAO↑GO↓	12
GO↓	7
FAO↑GO↑	6
FAO↓GO↓	2

*FAO* fatty acid oxidation, *GO* glucose oxidation, *↑ or ↓* increased or decreased.

### Meta-analysis

Meta-analysis of all included studies yields a positive pooled effect size (Hedges’ *g* = 0.69; 95% CI: 0.47–0.92; *p* < 0.001; Supplementary Fig. 1). However, according to the Q-test, there is substantial heterogeneity (Q(348) = 1227.60, *p* < 0.001, σ^2^ = 1.14, I^2^ = 80.96; Supplementary Fig. 1). Therefore, we performed meta-regression considering metabolic change and disease model as moderators. While the various disease models did not account for heterogeneity, meta-regression for metabolic change reduced the total amount of heterogeneity (σ^2^) by 51.52%.

### Interventions associated with increased fatty acid oxidation

**Fig. 2:**
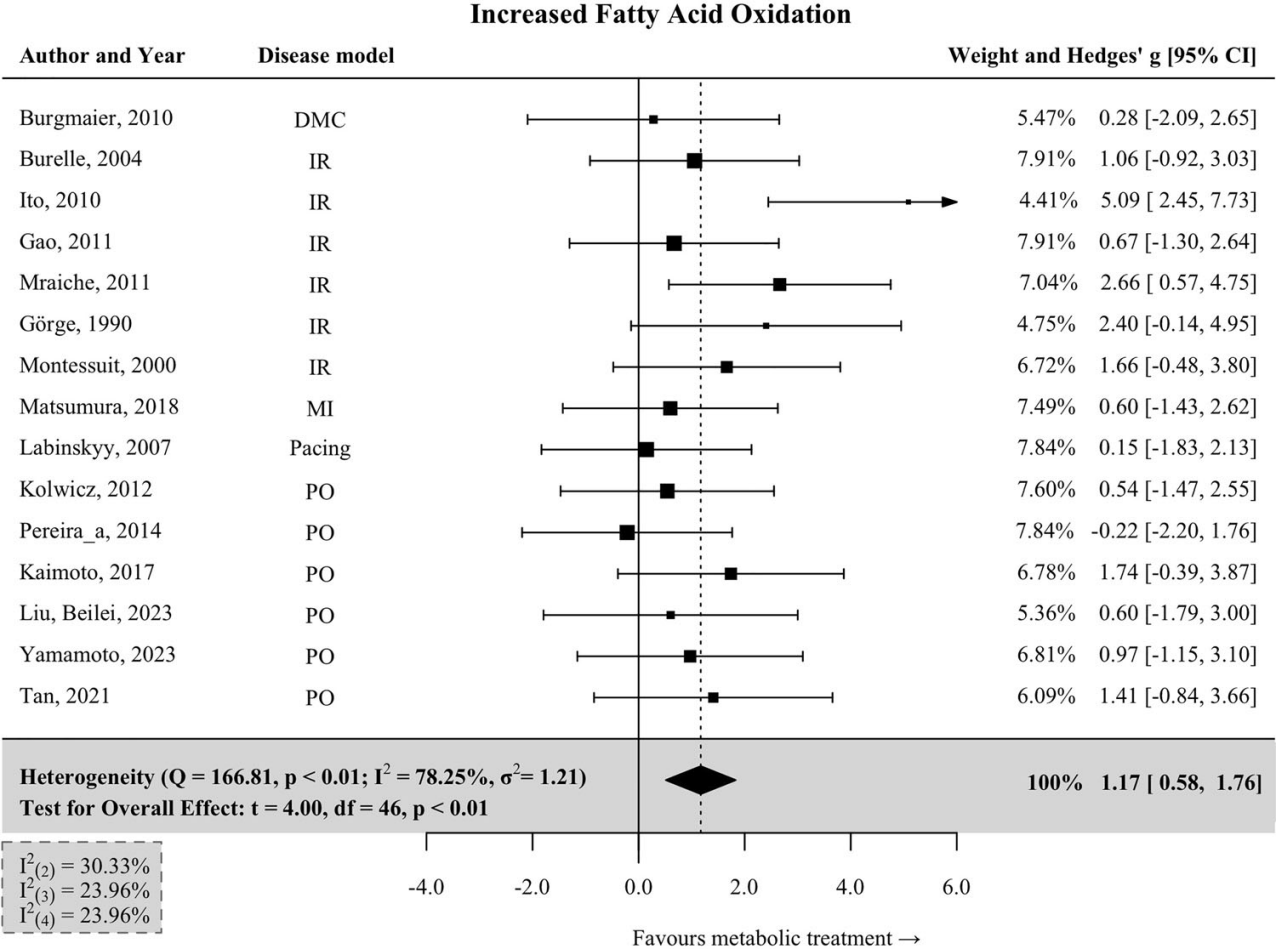
Interventions associated with increased cardiac fatty acid oxidation. Data are presented as Hedges’ g, which is calculated using a multivariate meta-analysis to enclose all measures of heart function reported in each study. Bars represent 95% CI. Tests for the overall effect and confidence intervals are based on the Knapp and Hartung method. Individual box sizes are determined by their weight in the meta-analysis model. Number of control (*n* = 127) and intervention group (*n* = 133) animals. The components of heterogeneity across levels are quantified by partitioned I^2^-statistics: (2) between-outcomes; (3) between-interventions; (4) between studies. IR ischemia-reperfusion, PO pressure overload, MI myocardial infarction, DMC diabetic cardiomyopathy, Pacing pacing-induced heart failure.

Fifteen interventions associated with increased fatty acid oxidation (FAO↑) were pooled in meta-analysis. We found that these treatments led to remarkable improvement of cardiac function (Hedges’ *g* = 1.17; 95% CI: 0.58–1.76; *p* < 0.001; Fig. 2). Study heterogeneity was substantial in this group (I^2^ = 78.25%). The models of HF mostly consisted of ischemia-reperfusion (*n* = 6) and pressure-overload (*n* = 6).

### Interventions associated with decreased fatty acid oxidation

**Fig. 3:**
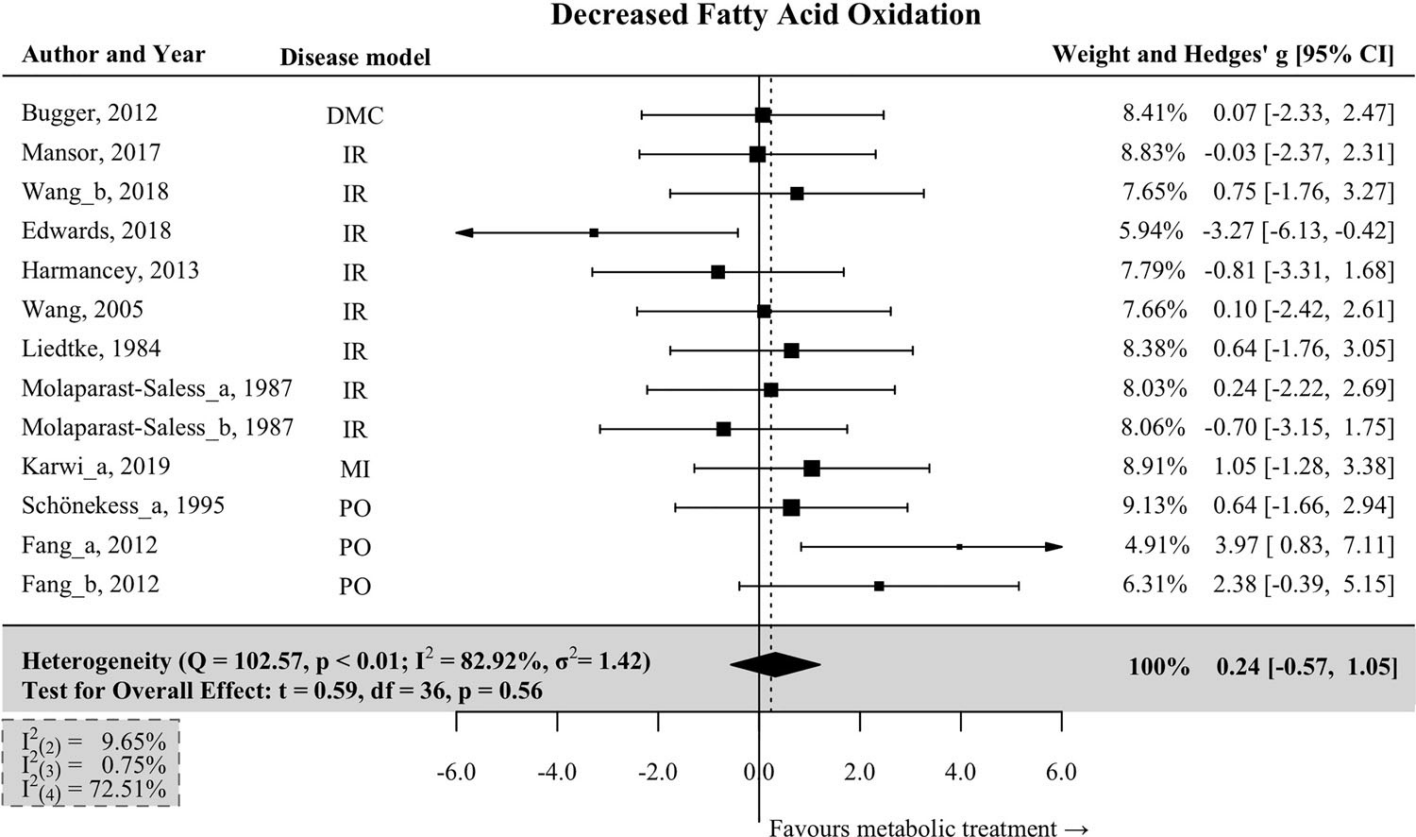
Interventions associated with decreased cardiac fatty acid oxidation. Data are presented as Hedges’ g, which is calculated using a multivariate meta-analysis to enclose all measures of heart function reported in each study. Bars represent 95% CI. Tests for the overall effect and confidence intervals are based on the Knapp and Hartung method. Individual box sizes are determined by their weight in the meta-analysis model. Number of control (*n* = 92) and intervention group (*n* = 88) animals. The components of heterogeneity across levels are quantified by partitioned I^2^-statistics: (2) between-outcomes; (3) between-interventions; (4) between studies. IR ischemia-reperfusion, PO pressure overload, MI myocardial infarction, DMC diabetic cardiomyopathy.

We found thirteen interventions resulting in a decreased rate of cardiac fatty acid oxidation (FAO ↓ ). The pooled effect size was small and not statistically significant (Hedges’ *g* = 0.24; 95% CI: −0.57–1.05; *p* = 0.557; Fig. 3). This subgroup exhibited high heterogeneity, with an I^2^ value of 82.92%, and included several different HF models.

### Interventions associated with increased glucose oxidation

**Fig. 4:**
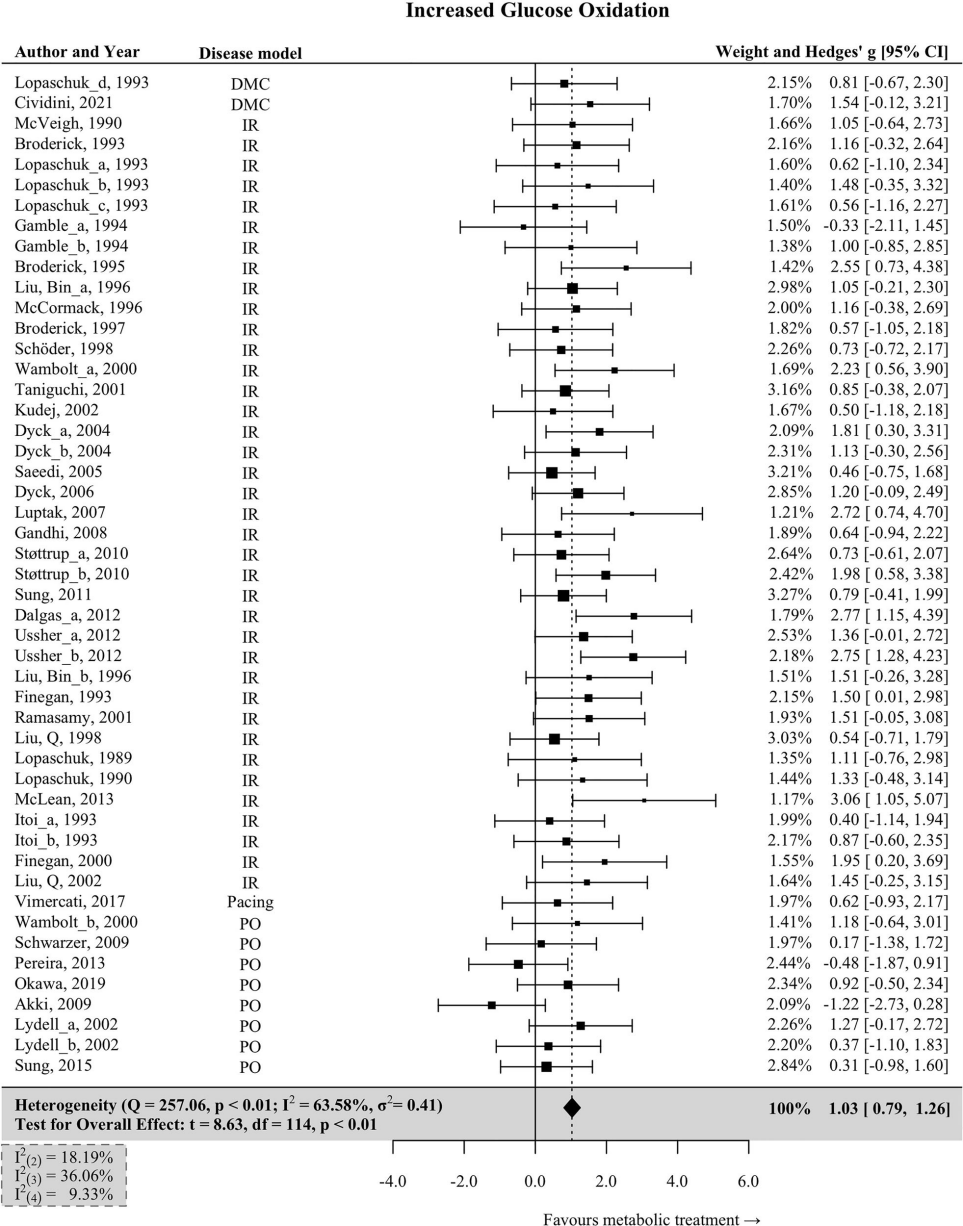
Interventions associated with increased cardiac glucose oxidation. Data are presented as Hedges’ g, which is calculated using a multivariate meta-analysis to enclose all measures of heart function reported in each study. Bars represent 95% CI. Tests for the overall effect and confidence intervals are based on the Knapp and Hartung method. Individual box sizes are determined by their weight in the meta-analysis model. Number of control (*n* = 393) and intervention group (*n* = 418) animals. The components of heterogeneity across levels are quantified by partitioned I^2^-statistics: (2) between-outcomes; (3) between-interventions; (4) between studies. IR ischemia-reperfusion, PO pressure overload, DMC diabetic cardiomyopathy, Pacing pacing-induced heart failure.

Forty-nine interventions leading to increased cardiac glucose oxidation (GO↑) were identified. The pooled effect size was large and significant (Hedges’ *g* = 1.03; 95% CI: 0.79–1.26; *p* < 0.001; Fig. 4), indicating a marked improvement of heart function. The Q-test suggests only moderate heterogeneity (I^2^ = 63.58), with ischemia-reperfusion (*n* = 38) as the predominant animal model of HF.

The results for interventions associated with decreased myocardial glucose oxidation (GO↓) were not analysed due to the limited number of studies (*n* = 7).

### Concomitant modulation of fatty acid oxidation and glucose oxidation

**Fig. 5:**
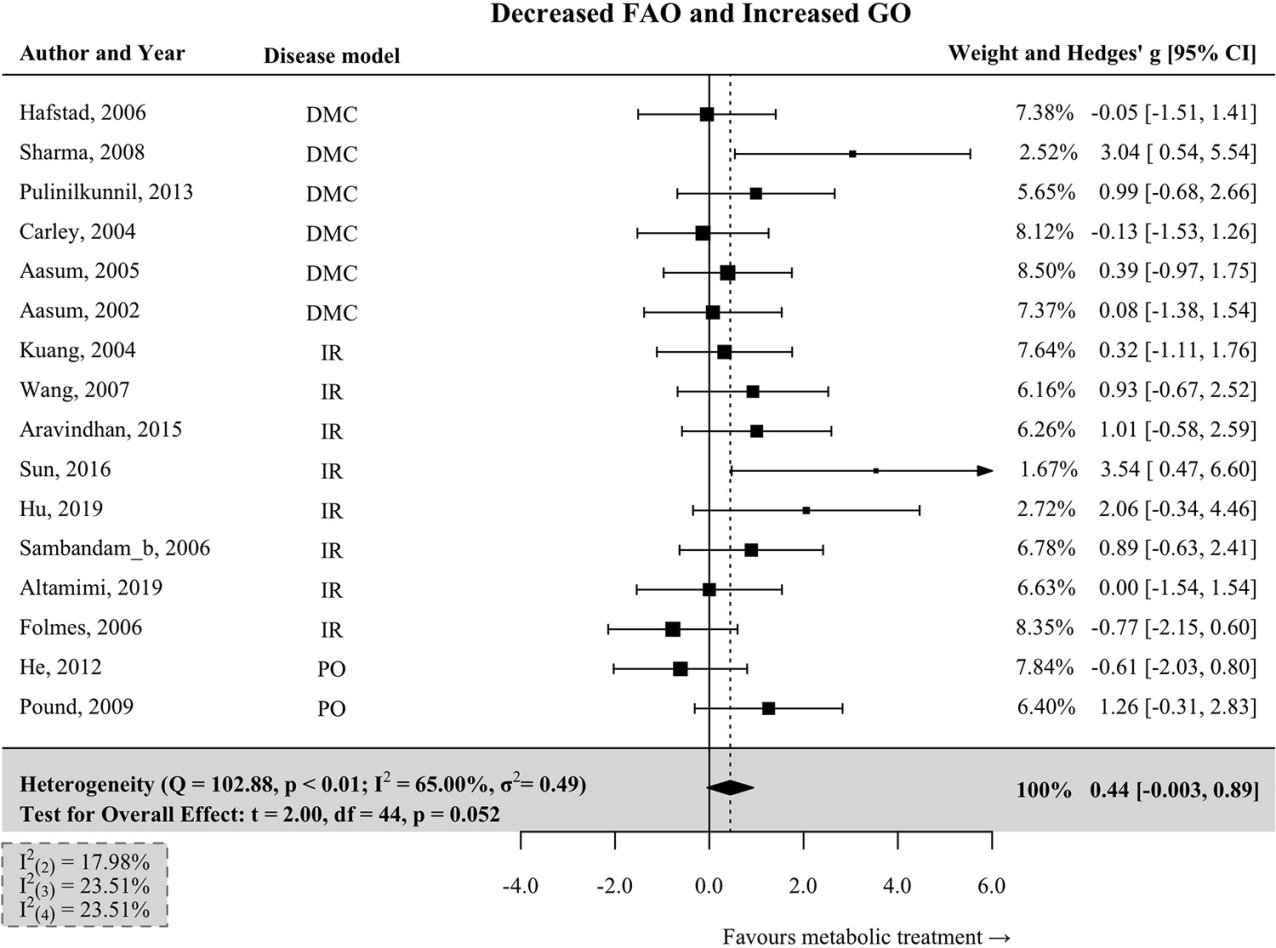
Interventions associated with decreased cardiac FAO and increased cardiac GO. Data are presented as Hedges’ g, which is calculated using a multivariate meta-analysis to enclose all measures of heart function reported in each study. Bars represent 95% CI. Tests for the overall effect and confidence intervals are based on the Knapp and Hartung method. Individual box sizes are determined by their weight in the meta-analysis model. Number of control (*n* = 147) and intervention group (*n* = 147) animals. The components of heterogeneity across levels are quantified by partitioned I^2^-statistics: (2) between-outcomes; (3) between-interventions; (4) between studies. FAO fatty acid oxidation, GO glucose oxidation, IR ischemia-reperfusion, PO pressure overload, DMC diabetic cardiomyopathy.

We also included interventions associated with concomitant changes in cardiac glucose and fatty acid oxidation. Approaches inhibiting fatty acid oxidation and enhancing myocardial glucose oxidation (FAO↓GO↑) resulted in a moderately positive effect size (Hedges’ *g* = 0.44; 95% CI: −0.003–0.89; *p* = 0.052; Fig. 5), which is not significant. We observed a moderate degree of heterogeneity in this group (I^2^ = 65.00%), with primarily diabetic (*n* = 6) and ischemia-reperfusion (*n* = 8) HF models.

The results of the twelve interventions reducing the rate of myocardial glucose oxidation and increasing fatty acid oxidation (FAO↑GO↓) showed no effect or significance (Hedges’ *g* = −0.03; 95% CI: −0.77–0.71; *p* = 0.945; Supplementary Fig. 2). Here, the outcome was substantially heterogeneous (I^2^ = 78.73%), and all five possible disease models were used equally. We did not analyse FAO↑GO↑ (*n* = 6) and FAO↓GO↓ (*n* = 2) because of the small group size.

### Publication bias and small-study effects

**Fig. 6:**
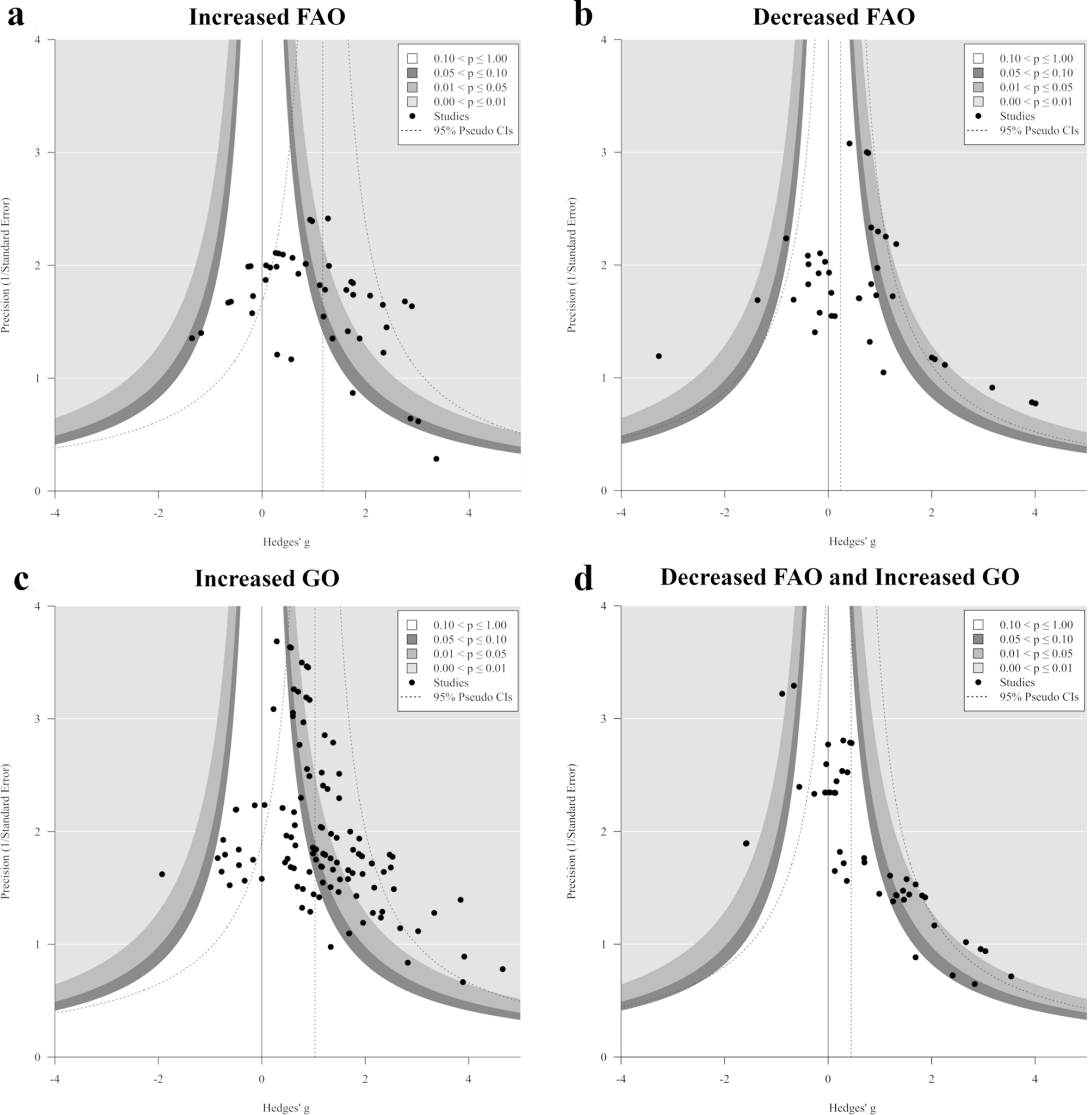
Contour-enhanced funnel plots for subgroups of metabolic change. **a** increased cardiac fatty acid oxidation (FAO); **b** decreased cardiac FAO; **c** increased cardiac glucose oxidation (GO); **d** decreased FAO and increased cardiac GO. The plot shows the observed effect sizes against the corresponding inverse standard errors. Contours of significance are centred around null, and dotted pseudo 95% confidence interval (CI) guidelines are depicted around the random-effects pooled estimate.

Funnel plot analysis revealed no asymmetry for the primary outcomes (Fig. 6, Supplementary Fig. 3). Except for FAO↓GO↑, the contour-enhanced funnel plots show that the effect sizes fan out in a symmetrical funnel shape around the pooled estimate and inside the 95% pseudo confidence intervals for every group of metabolic change. The independent contours of statistical significance also indicate that studies are distributed in areas of both low and high significance. Egger’s regression analysis showed that small study effects were present only in the FAO↓GO↑ group (Fig. 6d, *p* < 0.001), suggesting the possibility of missing publications in areas of low significance. Detailed results of Egger’s test are provided in Supplementary Table 2.

### Assessment of bias and study quality

Studies achieved a median score of 3 out of 8 possible points when evaluated for reporting quality using the CAMARADES checklist. With the help of the SYRCLE risk of bias tool, we identified 7 studies as high, 40 studies as low, and 73 as unknown in attrition bias. In the evaluation of selection bias, we found that among the 120 studies examined, 28 employed randomisation, while only 2 implemented allocation concealment (Supplementary Data 2).

### Sensitivity analysis

The sensitivity analysis was conducted to evaluate the robustness of the effect of metabolic modulation on heart function. In the leave-one-out analysis, each study was iteratively excluded from the meta-analysis to observe the influence on the overall effect estimate. In all tests, FAO↑ consistently showed strong effects (Hedges’ g: 0.99–1.29) with highly significant p-values (below 0.01), whereas FAO↓ did not show statistical significance in any iteration and remained universally neutral. GO↑ maintained significant effects (*p* < 0.001) with consistently large estimates (Hedges’ *g* > 1.00). The effect size for the concomitant change FAO↓GO↑ remained moderate (from 0.38 to 0.56) with p-values between 0.03 and 0.11. No changes were observed in sensitivity analysis for FAO↑GO↓. For visual assessment, the results are depicted as forest plots in Supplementary Figs. 4–8.

## Discussion

In this meta-analysis, we assessed the effect of treatments associated with changes in cardiac metabolism across various models of HF. Our findings suggest that increasing myocardial FAO or GO may significantly restore cardiac function (Figs. 2 and  4). These metabolic treatment strategies showed remarkably large effect sizes (FAO↑ = 1.17; GO↑ = 1.03) according to the common interpretation of Hedges’ g to refer to effect sizes as large (*g* > 0.8)^40^. Notably, studies measuring decreased cardiac FAO did not show improvements in cardiac function (Fig. 3). Even in interventions in which a decreased FAO was accompanied by a concomitant increase in GO, we found only a moderate and nonsignificant effect (Fig. 5). Our findings on the effects of inhibiting FAO disagree with the current notion of metabolic modulation in HF, which recommends inhibiting cardiac FAO to improve heart function^8,24^.

The rationale for inhibiting cardiac FAO is based on the principle of the Randle cycle, which states that suppressing FAO in the heart can lead to a compensatory increase in GO^41^. This switch may have an oxygen-sparing effect resulting in higher cardiac efficiency^42–46^. However, it is unclear whether this mechanism applies to the failing heart that undergoes profound metabolic remodelling^47^. Because both cardiac FAO and GO are impaired in HF, further suppression of FAO may not be sufficiently compensated by GO and therefore compromise energy production^23^. In contrast, our results suggest that enhancing cardiac FAO, similarly to GO, may considerably improve heart function by counteracting energy deficiency.

The notion of inhibiting cardiac FAO to treat HF is apparently supported by the fact that some FAO inhibitors such as trimetazidine, etomoxir, and perhexiline have shown therapeutic potential^9–11^. However, it is unclear if the putative inhibition of cardiac FAO accounted for the functional improvements since FAO was not always measured. Therefore, we selected only studies reporting oxidation rates in diseased cardiac tissues. This approach enabled the identification of actual metabolic alterations resulting from therapeutic interventions, regardless of preconceived expectations. Interestingly, we observed that the rate of FAO was not inhibited in several cases of HF treated with FAO inhibitors^13–15,21^.

From a clinical perspective, our results are relevant by suggesting stimulation of cardiac FAO and GO as a specific target for developing new HF treatments. Previous efforts have focused on inhibiting cardiac FAO leaving its activation largely unexplored. Although some fatty acid activators are available, for example: acetyl-CoA carboxylase inhibitors^48^, they have never been studied in the context of HF. With respect to the stimulation of cardiac GO, dichloroacetate (DCA) has been the most investigated agent so far. While DCA has been shown to improve cardiac work and reduce oxygen consumption in patients with advanced HF^49^, long-term clinical trials are lacking, partly because of the risk of neuropathy^50^.

While meta-analyses frequently pool the results of a single cardiac function parameter (e.g., ejection fraction) and perform a univariate analysis, we chose to assess multiple measures via a multilevel model. This methodology uses additional levels of meta-analysis to address the dependent effect sizes within and between studies, accounting for their correlations^51^. Consequently, it offered certain advantages. First, it allows for the assessment of not only systolic function but also changes in diastolic, morphological, and histological parameters. Second, we were able to analyse a total of 103 publications including those that conducted only ex vivo experiments. While it may be limiting that only 38 over 103 studies reported data from echocardiography, many studies would have been overlooked if ejection fraction, for instance, had been used as the only functional outcome.

Publication bias is a serious problem in systematic reviews and meta-analyses because it tends to inflate effect size estimates^52^. However, the impact of publication bias on the main results may be minimal (Fig. 6, Supplementary Table 2). Publication bias was only suggested in the FAO↓GO↑ group, possibly due to the prevailing notion that recommends reducing myocardial FAO and increasing GO in HF. Therefore, the effect size may be overestimated in this group.

When interpreting the results of our study, it is important to consider further aspects. First, we decided to exclude data from human studies for the following reason. Our preliminary search failed to identify human studies with direct measurements of cardiac substrate oxidation. However, this issue is essential because the rate of FAO may be unaffected in several cases of HF treated with FAO inhibitors^13–15,53^. Second, changes in cardiac GO and FAO and therefore the effectiveness of metabolic modulation may vary depending on the etiologies of HF^22,47^. Although we included all established models of HF, IR and PO were most commonly represented. Therefore, our findings may best apply to IR and PO models, while the impact of metabolic modulation on other models requires further validation. Third, our inclusive approach (i.e., different models, outcomes, interventions) may have introduced additional sources of variability resulting in high heterogeneity. Thus, the I^2^ statistic remained above 70% in every metabolic subgroup except for GO↑ and FAO↓GO↑, where IR is the predominant disease model. We suspect that differences among disease models may play a role. Additionally, the coexistence of models with reduced and preserved ejection fraction may contribute to the residual heterogeneity in the subgroups. However, a further regression analysis was not possible because of the low number of studies within each subgroup (*n* < 10).

## Conclusions

Our data underscore cardiac metabolism as a potential target to treat heart failure. Specifically, they suggest that stimulating either myocardial FAO or GO may considerably improve cardiac function. Furthermore, they question the current notion that inhibition of cardiac FAO is protective.

## Supplementary information

Supplementary Information

Description of Additional Supplementary Files

Supplementary Data 1

Supplementary Data 2

Supplementary Data 3

Supplementary Data 4

Reporting Summary

## Data Availability

This meta-analysis is based on data extracted from publicly available studies. All data generated or analysed during this study are available within the paper and its supplementary files. The characteristics of all included studies can be found in Supplementary Data 1. The source data for the assessment of bias and study quality can be found in Supplementary Data 2. The extracted numerical data underlying the forest plots and regression analyses can be found in Supplementary Data 3. The source data for the contour-enhanced funnel plots in Fig. 6 can be found in Supplementary Data 4.

## Abbreviations

DMC: diabetic cardiomyopathy
DCA: dichloroacetate
FAO: fatty acid oxidation
GO: glucose oxidation
HF: heart failure
HFpEF: heart failure with preserved ejection fraction
IR: ischemia-reperfusion
MI: myocardial infarction
PO: pressure overload
PRISMA: Preferred Reporting Items for Systematic Reviews and Meta-Analyses

## Code availability

The code used for the meta-analysis is available at: https://github.com/detoxtom/Meta-Analysis-on-Metabolic-Modulation-in-HF, and via Zenodo^54^.
