# Supplementary material for: Modulation of cardiac fatty acid or glucose oxidation to treat heart failure in preclinical models: a systematic review and meta-analysis
Source: Commun Med (Lond). 2025 Jun 4;5:213. doi: 10.1038/s43856-025-00924-5 (PMC12134058; doi:10.1038/s43856-025-00924-5)
Supplement: Supplementary file 1 — Supplementary Information [file 43856_2025_924_MOESM1_ESM.pdf]

SUPPLEMENTAL MATERIAL

Modulation of cardiac fatty acid or glucose oxidation to treat heart failure in preclinical models: A systematic review and meta-analysis

Tom Fischer[1], Christina Schenkl[2], Estelle Heyne[2], Peter Schlattmann[3], Torsten Doenst[2], P. Christian Schulze[1], T. Dung Nguyen[1]

[1] Department of Medicine I (Cardiology, Angiology, Intensive Care Medicine), Jena University Hospital, Friedrich Schiller University Jena, Am Klinikum 1, 07747 Jena, Germany.
[2] Department of Cardiothoracic Surgery, Jena University Hospital, Friedrich Schiller University Jena, Am Klinikum 1, 07747 Jena, Germany
[3] Institute of Medical Statistics, Computer and Data Sciences, Jena University Hospital, Friedrich Schiller University Jena, Bachstraße 18, 07743 Jena, Germany.

Contents

Supplementary Methods
Supplementary Tables 1-2
Supplementary Figures 1-8
References

Supplementary Methods

Detailed Search Strategy

Ovid Medline – 03/02/2024

Ovid MEDLINE(R) ALL <1946 to March 02, 2024>

1 (exp Heart/ or heart.tw. or exp Myocardium/ or myocard*.tw. or exp Myocytes, Cardiac/ or cardiomyocyte*.tw. or exp Heart Ventricles/ or left ventric*.tw. or exp Ventricular Function/ or (ventric* adj3 function*).tw. or (cardi* adj3 function*).tw. or exp Heart Diseases/ or exp Heart Injuries/ or exp Cardiomyopathy/ or cardiomyopath*.tw. or exp Heart Failure/ or (cardi* adj3 fail*).tw. or exp Ventricular Dysfunction/ or (cardi* adj3 dysfunction*).tw.) and (fat* oxid* or fat* acid* oxid* or beta oxid* or FA-Oxid* or FA-Metabolism or Palmit* Oxid* or Olea* Oxid* or Fat* acid* metabolism or carbohydrat* oxid* or glucose oxid* or pyruvate oxid* or glucose metabolism or metabolic modulat*).tw.

Web of Science – 03/02/2024

((TI=(heart) OR TI= (myocard*) OR TI=(cardiomyocyte*) OR TI=(left ventric*) OR TI=(cardiomyopath*) OR TI=(cardi* NEAR/3 fail*) OR TI=(cardi* NEAR/3 function*) OR TI=(ventric* NEAR/3 function*) OR TI=(cardi* NEAR/3 dysfunction*) OR TI=(ventric* NEAR/3 dysfunction*)) AND (TI=("fat* acid* oxid*") OR TI=("fat* oxid*") OR TI=("beta oxid*") OR TI=("FA oxid*") OR TI=("FA Metabolism") OR TI=("Palmit* Oxid*") OR TI=("Olea* Oxid*") OR TI=("Fat* acid* metabolism*") OR TI=("carbohydrat* oxid*") OR TI=("glucose oxid*") OR TI=("pyruvate oxid*") OR TI=(“glucose metabolism”) OR TI=(“metabolic modulat*”))) OR ((AB=(heart) OR AB= (myocard*) OR AB=(cardiomyocyte*) OR AB=(left ventric*) OR AB=(cardiomyopath*) OR AB=(cardi* NEAR/3 fail*) OR AB=(cardi* NEAR/3 function*) OR AB=(ventric* NEAR/3 function*) OR AB=(cardi* NEAR/3 dysfunction*) OR AB=(ventric* NEAR/3 dysfunction*)) AND (AB=("fat* acid* oxid*") OR AB=("fat* oxid*") OR AB=("beta oxid*") OR

AB=("FA oxid*") OR AB=("FA Metabolism") OR AB=("Palmit* Oxid*") OR AB=("Olea* Oxid*") OR AB=("Fat* acid* metabolism*") OR AB=("carbohydrat* oxid*") OR AB=("glucose oxid*") OR AB=("pyruvate oxid*") OR AB=(“glucose metabolism”) OR AB=(“metabolic modulat*”)))

<u>Scopus - 03/02/2024</u>

TITLE-ABS ((heart OR myocard* OR cardiomyocyte* OR (left AND ventric*) OR cardiomyopath* OR (cardi* W/3 fail*) OR (cardi* W/3 function*) OR (ventric* W/3 function*) OR (cardi* W/3 dysfunction*) OR (ventric* W/3 dysfunction*)) AND ("fat* oxid*" OR "fat* acid* oxid*" OR "beta oxid*" OR "FA-Oxid*" OR "FA-Metabolism" OR "Palmit* Oxid*" OR "Olea* Oxid*" OR "Fat* acid* metabolism" OR "carbohydrate* oxid*" OR "glucose oxid*" OR "pyruvate oxid*" OR “glucose metabolism” OR “metabolic modulat*”))

Metabolic Change	Estimate	p-value	t-value	df	95% Confidence interval
FAO↑	1.04	7.28×10^{-5}	4.41	41	0.56 - 1.52
FAO↓	0.21	0.696	0.39	29	-0.86 - 1.27
GO↑	1.08	2.94×10^{-13}	8.31	109	0.82 - 1.34
GO↑FAO↓	0.44	0.051	4.58	44	-0.00 - 0.89
GO↓FAO↑	-0.06	0.889	-0.41	47	-0.92 - 0.80

Supplementary Table 1: Subgroup analysis for studies with rodents.

Estimates presented as Hedges' g. Results of multivariate meta-analysis when only rodent animals are considered. FAO: fatty acid oxidation, GO: glucose oxidation, ↑ or ↓: increased or decreased, df: degrees of freedom

Metabolic Change	Intercept	p-value	t-value	df	95% Confidence interval
FAO↑	1.61	0.322	1.00	45	0.54 - 2.68
FAO↓	-0.12	0.434	0.79	35	-1.34 - 1.10
GO↑	0.89	0.504	0.67	113	0.40 - 1.37
GO↑FAO↓	-0.83	4.00×10^{-5}	4.58	43	-1.39 to -0.274
GO↓FAO↑	0.28	0.661	0.44	52	-1.69 - 1.12

Supplementary Table 2: Regression test for funnel plot asymmetry.

FAO: fatty acid oxidation, GO: glucose oxidation, ↑ or ↓: increased or decreased, df: degrees of freedom

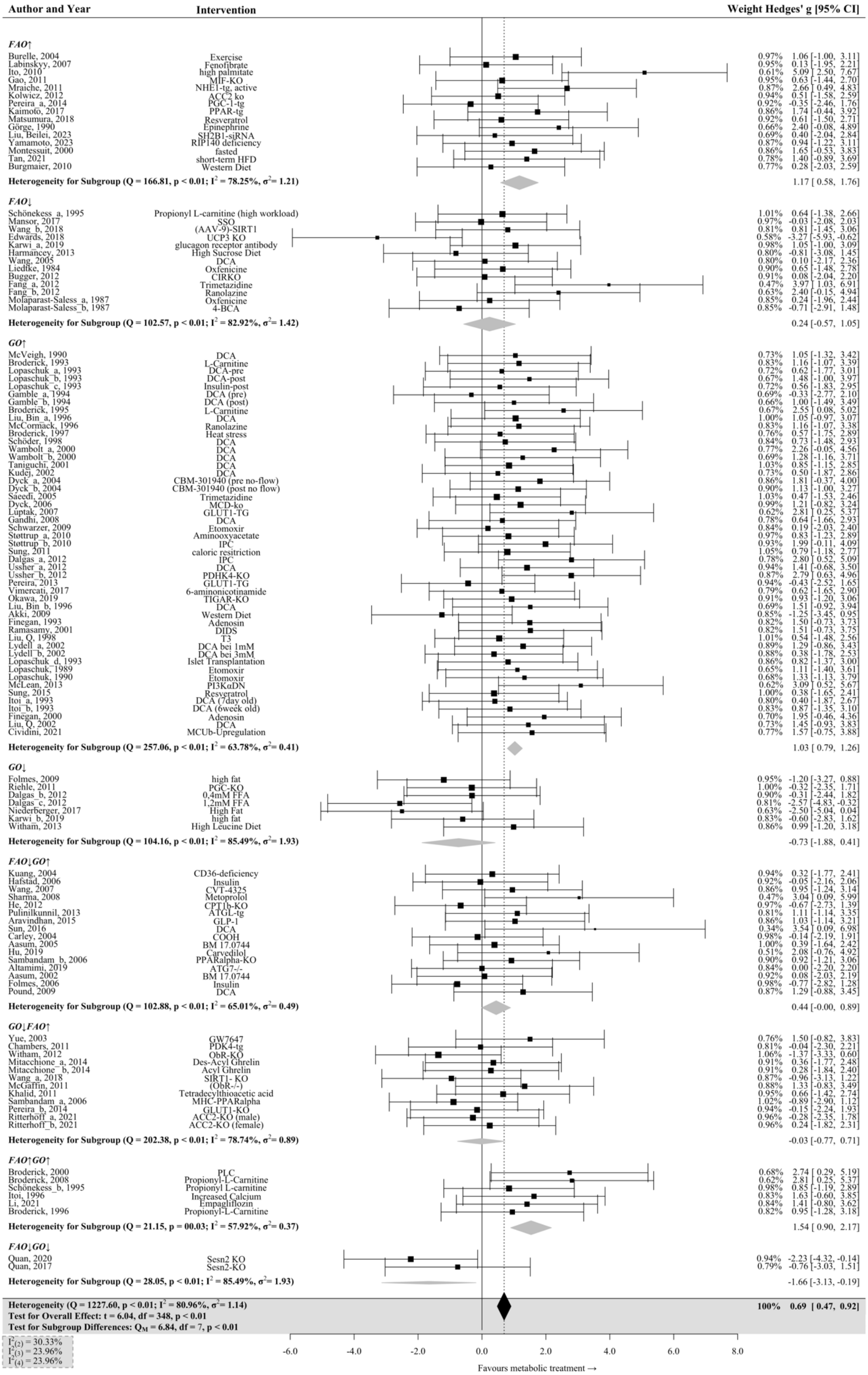
Author and Year
Intervention
Weight Hedges' g [95% CI]
FAO↑
Burelle, 2004 Exercise 0.97% 1.06 [-1.00, 3.11]
Labinskyy, 2007 Fenofibrate 0.95% 0.13 [-1.95, 2.21]
Ito, 2010 high palmitate 0.61% 5.09 [2.50, 7.67]
Gao, 2011 MIF-KO 0.95% 0.63 [-1.44, 2.70]
Mraiche, 2011 NHE1-tg, active 0.87% 2.66 [0.49, 4.83]
Kolwicz, 2012 ACC2 ko 0.94% 0.51 [-1.58, 2.59]
Pereira_a, 2014 PGC-1-tg 0.92% -0.35 [-2.46, 1.76]
Kaimoto, 2017 PPAR-tg 0.86% 1.74 [-0.44, 3.92]
Matsumura, 2018 Resveratrol 0.92% 0.61 [-1.50, 2.71]
Görge, 1990 Epinephrine 0.66% 2.40 [-0.08, 4.89]
Liu, Beilei, 2023 SH2B1-siRNA 0.69% 0.40 [-2.04, 2.84]
Yamamoto, 2023 RIP140 deficiency 0.87% 0.94 [-1.22, 3.11]
Montessuit, 2000 fasted 0.86% 1.65 [-0.53, 3.83]
Tan, 2021 short-term HFD 0.78% 1.40 [-0.89, 3.69]
Burgmaier, 2010 Western Diet 0.77% 0.28 [-2.03, 2.59]
Heterogeneity for Subgroup (Q = 166.81, p < 0.01; I^2 = 78.25%, σ^2= 1.21) 1.17 [0.58, 1.76]
FAO↓
Schönekess_a, 1995 Propionyl L-carnitine (high workload) 1.01% 0.64 [-1.38, 2.66]
Mansor, 2017 SSO 0.97% -0.03 [-2.08, 2.03]
Wang_b, 2018 (AAV-9)-SIRT1 0.81% 0.81 [-1.45, 3.06]
Edwards, 2018 UCP3 KO 0.58% -3.27 [-5.93, -0.62]
Karwi_a, 2019 glucagon receptor antibody 0.98% 1.05 [-1.00, 3.09]
Harmancey, 2013 High Sucrose Diet 0.80% -0.81 [-3.08, 1.45]
Wang, 2005 DCA 0.80% 0.10 [-2.17, 2.36]
Liedtke, 1984 Oxfenicine 0.90% 0.65 [-1.48, 2.78]
Bugger, 2012 CIRKO 0.91% 0.08 [-2.04, 2.20]
Fang_a, 2012 Trimetazidine 0.47% 3.97 [1.03, 6.91]
Fang_b, 2012 Ranolazine 0.63% 2.40 [-0.15, 4.94]
Molaparast-Saless_a, 1987 Oxfenicine 0.85% 0.24 [-1.96, 2.44]
Molaparast-Saless_b, 1987 4-BCA 0.85% -0.71 [-2.91, 1.48]
Heterogeneity for Subgroup (Q = 102.57, p < 0.01; I^2 = 82.92%, σ^2= 1.42) 0.24 [-0.57, 1.05]
GO↑
McVeigh, 1990 DCA 0.73% 1.05 [-1.32, 3.42]
Broderick, 1993 L-Carnitine 0.83% 1.16 [-1.07, 3.39]
Lopaschuk_a, 1993 DCA-pre 0.72% 0.62 [-1.77, 3.01]
Lopaschuk_b, 1993 DCA-post 0.67% 1.48 [-1.00, 3.97]
Lopaschuk_c, 1993 Insulin-post 0.72% 0.56 [-1.83, 2.95]
Gamble_a, 1994 DCA (pre) 0.69% -0.33 [-2.77, 2.10]
Gamble_b, 1994 DCA (post) 0.66% 1.00 [-1.49, 3.49]
Broderick, 1995 L-Carnitine 0.67% 2.55 [0.08, 5.02]
Liu, Bin_a, 1996 DCA 1.00% 1.05 [-0.97, 3.07]
McCormack, 1996 Ranolazine 0.83% 1.16 [-1.07, 3.38]
Broderick, 1997 Heat stress 0.76% 0.57 [-1.75, 2.89]
Schöder, 1998 DCA 0.84% 0.73 [-1.48, 2.93]
Wambolt_a, 2000 DCA 0.77% 2.26 [-0.05, 4.56]
Wambolt_b, 2000 DCA 0.69% 1.28 [-1.16, 3.71]
Taniguchi, 2001 DCA 1.03% 0.85 [-1.15, 2.85]
Kudej, 2002 DCA 0.73% 0.50 [-1.87, 2.86]
Dyck_a, 2004 CBM-301940 (pre no-flow) 0.86% 1.81 [-0.37, 4.00]
Dyck_b, 2004 CBM-301940 (post no flow) 0.90% 1.13 [-1.00, 3.27]
Saeedi, 2005 Trimetazidine 1.03% 0.47 [-1.53, 2.46]
Dyck, 2006 MCD-ko 0.99% 1.21 [-0.82, 3.24]
Luptak, 2007 GLUT1-TG 0.62% 2.81 [0.25, 5.37]
Gandhi, 2008 DCA 0.78% 0.64 [-1.66, 2.93]
Schwarzer, 2009 Etomoxir 0.84% 0.19 [-2.03, 2.40]
Støttrup_a, 2010 Aminooxyacetate 0.97% 0.83 [-1.23, 2.89]
Støttrup_b, 2010 IPC 0.93% 1.99 [-0.11, 4.09]
Sung, 2011 caloric resitriction 1.05% 0.79 [-1.18, 2.77]
Dalgas_a, 2012 IPC 0.78% 2.80 [0.52, 5.09]
Ussher_a, 2012 DCA 0.94% 1.41 [-0.68, 3.50]
Ussher_b, 2012 PDHK4-KO 0.87% 2.79 [0.63, 4.96]
Pereira, 2013 GLUT1-TG 0.94% -0.43 [-2.52, 1.65]
Vimercati, 2017 6-aminonicotinamide 0.79% 0.62 [-1.65, 2.90]
Okawa, 2019 TIGAR-KO 0.91% 0.93 [-1.20, 3.06]
Liu, Bin_b, 1996 DCA 0.69% 1.51 [-0.92, 3.94]
Akki, 2009 Western Diet 0.85% -1.25 [-3.45, 0.95]
Finegan, 1993 Adenosin 0.82% 1.50 [-0.73, 3.73]
Ramasamy, 2001 DIDS 0.82% 1.51 [-0.73, 3.75]
Liu, Q, 1998 T3 1.01% 0.54 [-1.48, 2.56]
Lydell_a, 2002 DCA bei 1mM 0.89% 1.29 [-0.86, 3.43]
Lydell_b, 2002 DCA bei 3mM 0.88% 0.38 [-1.78, 2.53]
Lopaschuk_d, 1993 Islet Transplantation 0.86% 0.82 [-1.37, 3.00]
Lopaschuk, 1989 Etomoxir 0.65% 1.11 [-1.40, 3.61]
Lopaschuk, 1990 Etomoxir 0.68% 1.33 [-1.13, 3.79]
McLean, 2013 PI3KαDN 0.62% 3.09 [0.52, 5.67]
Sung, 2015 Resveratrol 1.00% 0.38 [-1.65, 2.41]
Itoi_a, 1993 DCA (7day old) 0.80% 0.40 [-1.87, 2.67]
Itoi_b, 1993 DCA (6week old) 0.83% 0.87 [-1.35, 3.10]
Finegan, 2000 Adenosin 0.70% 1.95 [-0.46, 4.36]
Liu, Q, 2002 DCA 0.73% 1.45 [-0.93, 3.83]
Cividini, 2021 MCUb-Upregulation 0.77% 1.57 [-0.75, 3.88]
Heterogeneity for Subgroup (Q = 257.06, p < 0.01; I^2 = 63.78%, σ^2= 0.41) 1.03 [0.79, 1.26]
GO↓
Folmes, 2009 high fat 0.95% -1.20 [-3.27, 0.88]
Riehle, 2011 PGC-KO 1.00% -0.32 [-2.35, 1.71]
Dalgas_b, 2012 0,4mM FFA 0.90% -0.31 [-2.44, 1.82]
Dalgas_c, 2012 1,2mM FFA 0.81% -2.57 [-4.83, -0.32]
Niederberger, 2017 High Fat 0.63% -2.50 [-5.04, 0.04]
Karwi_b, 2019 high fat 0.83% -0.60 [-2.83, 1.62]
Witham, 2013 High Leucine Diet 0.86% 0.99 [-1.20, 3.18]
Heterogeneity for Subgroup (Q = 104.16, p < 0.01; I^2 = 85.49%, σ^2= 1.93) -0.73 [-1.88, 0.41]
FAO↓GO↑
Kuang, 2004 CD36-deficiency 0.94% 0.32 [-1.77, 2.41]
Hafstad, 2006 Insulin 0.92% -0.05 [-2.16, 2.06]
Wang, 2007 CVT-4325 0.86% 0.95 [-1.24, 3.14]
Sharma, 2008 Metoprolol 0.47% 3.04 [0.09, 5.99]
He, 2012 CPT1b-KO 0.97% -0.67 [-2.73, 1.39]
Pulinilkunnil, 2013 ATGL-tg 0.81% 1.11 [-1.14, 3.35]
Aravindhan, 2015 GLP-1 0.86% 1.03 [-1.14, 3.21]
Sun, 2016 DCA 0.34% 3.54 [0.09, 6.98]
Carley, 2004 COOH 0.98% -0.14 [-2.19, 1.91]
Aasum, 2005 BM 17.0744 1.00% 0.39 [-1.64, 2.42]
Hu, 2019 Carvedilol 0.51% 2.08 [-0.76, 4.92]
Sambandam_b, 2006 PPARalpha-KO 0.90% 0.92 [-1.21, 3.06]
Altamimi, 2019 ATG7-/- 0.84% 0.00 [-2.20, 2.20]
Aasum, 2002 BM 17.0744 0.92% 0.08 [-2.03, 2.19]
Folmes, 2006 Insulin 0.98% -0.77 [-2.82, 1.28]
Pound, 2009 DCA 0.87% 1.29 [-0.88, 3.45]
Heterogeneity for Subgroup (Q = 102.88, p < 0.01; I^2 = 65.01%, σ^2= 0.49) 0.44 [-0.00, 0.89]
GO↓FAO↑
Yue, 2003 GW7647 0.76% 1.50 [-0.82, 3.83]
Chambers, 2011 PDK4-tg 0.81% -0.04 [-2.30, 2.21]
Witham, 2012 ObR-KO 1.06% -1.37 [-3.33, 0.60]
Mitacchione_a, 2014 Des-Acyl Ghrelin 0.91% 0.36 [-1.77, 2.48]
Mitacchione_b, 2014 Acyl Ghrelin 0.91% 0.28 [-1.84, 2.40]
Wang_a, 2018 SIRT1- KO 0.87% -0.96 [-3.13, 1.22]
McGaffin, 2011 (ObR-/-) 0.88% 1.33 [-0.83, 3.49]
Khalid, 2011 Tetradecylthioacetic acid 0.95% 0.66 [-1.42, 2.74]
Sambandam_a, 2006 MHC-PPARalpha 1.02% -0.89 [-2.90, 1.12]
Pereira_b, 2014 GLUT1-KO 0.94% -0.15 [-2.24, 1.93]
Ritterhoff_a, 2021 ACC2-KO (male) 0.96% -0.28 [-2.35, 1.78]
Ritterhoff_b, 2021 ACC2-KO (female) 0.96% 0.24 [-1.82, 2.31]
Heterogeneity for Subgroup (Q = 202.38, p < 0.01; I^2 = 78.74%, σ^2= 0.89) -0.03 [-0.77, 0.71]
FAO↑GO↑
Broderick, 2000 PLC 0.68% 2.74 [0.29, 5.19]
Broderick, 2008 Propionyl-L-Carnitine 0.62% 2.81 [0.25, 5.37]
Schönekess_b, 1995 Propionyl L-carnitine 0.98% 0.85 [-1.19, 2.89]
Itoi, 1996 Increased Calcium 0.83% 1.63 [-0.60, 3.85]
Li, 2021 Empagliflozin 0.84% 1.41 [-0.80, 3.62]
Broderick, 1996 Propionyl-L-Carnitine 0.82% 0.95 [-1.28, 3.18]
Heterogeneity for Subgroup (Q = 21.15, p = 00.03; I^2 = 57.92%, σ^2= 0.37) 1.54 [0.90, 2.17]
FAO↓GO↓
Quan, 2020 Sesn2 KO 0.94% -2.23 [-4.32, -0.14]
Quan, 2017 Sesn2-KO 0.79% -0.76 [-3.03, 1.51]
Heterogeneity for Subgroup (Q = 28.05, p < 0.01; I^2 = 85.49%, σ^2= 1.93) -1.66 [-3.13, -0.19]
Heterogeneity (Q = 1227.60, p < 0.01; I^2 = 80.96%, σ^2= 1.14) 100% 0.69 [0.47, 0.92]
Test for Overall Effect: t = 6.04, df = 348, p < 0.01
Test for Subgroup Differences: Q_M = 6.84, df = 7, p < 0.01
$I^2_{(2)}$ = 30.33%
$I^2_{(3)}$ = 23.96%
$I^2_{(4)}$ = 23.96%
-6.0 -4.0 -2.0 0.0 2.0 4.0 6.0 8.0
Favours metabolic treatment →

Supplementary Figure 1: Forest plot of all included studies[1-103]. Grouped by metabolic change**.** Data are presented as Hedges' g, which is calculated using multivariate meta-analysis to enclose all measures of heart function reported in each study. Bars represent 95% CI. Tests for the overall effect and confidence intervals are based on the Knapp and Hartung method. Individual box sizes are determined by their weight in the meta-analysis model. Number of control (n = 1027) and intervention group (n = 995) animals. The components of heterogeneity across levels are quantified by partitioned I^2-statistics: (2) between-outcomes; (3) between-interventions; (4) between studies. FAO: fatty acid oxidation, GO: glucose oxidation, ↑ or ↓: increased or decreased.

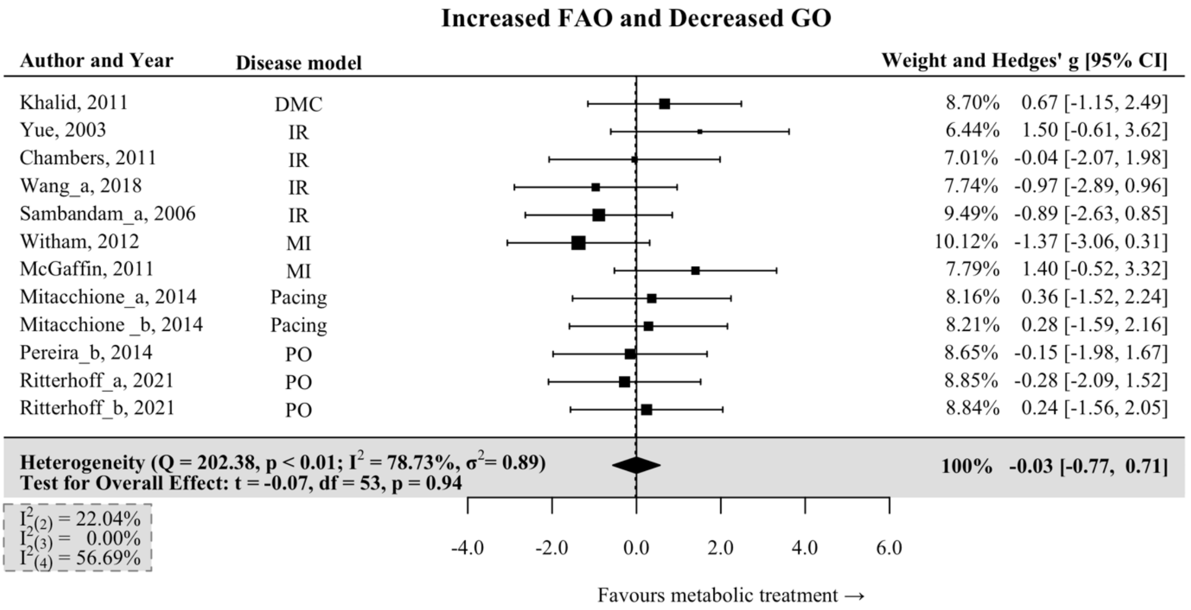

Supplementary Figure 2: Forest plot of interventions associated with increased cardiac fatty acid oxidation and decreased cardiac glucose oxidation (FAO↑GO↓). Data are presented as Hedges' g, which is calculated using a multivariate meta-analysis to enclose all measures of heart function reported in each study. Bars represent 95% CI. Tests for the overall effect and confidence intervals are based on the Knapp and Hartung method. Individual box sizes are determined by their weight in the meta-analysis model. Number of control and (n = 96) intervention group (n = 100) animals. The components of heterogeneity across levels are quantified by partitioned I^2-statistics: (2) between-outcomes; (3)

between-interventions; (4) between studies. IR: ischemia-reperfusion, PO: pressure overload, MI: myocardial infarction, DMC: diabetic cardiomyopathy, Pacing: pacing-induced heart failure.

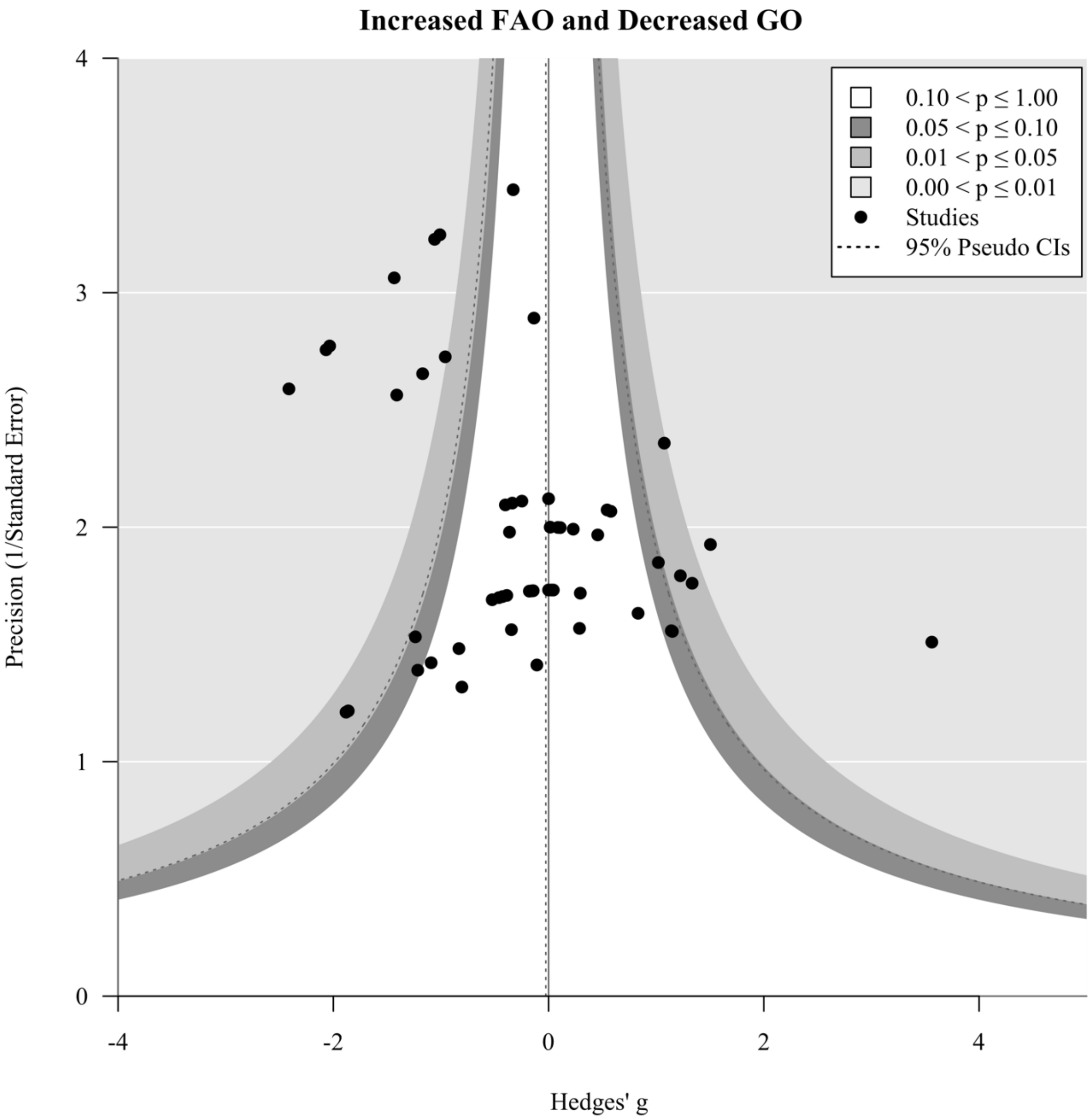

Supplementary Figure 3: **Contour-enhanced funnel plot of interventions associated with increased cardiac fatty acid oxidation and decreased cardiac glucose oxidation (FAO↑GO↓).** The plot shows the observed effect sizes against the corresponding inverse standard errors. Contours of significance are centered around null, and dotted pseudo 95% confidence interval (CI) guidelines are depicted around the random-effects pooled estimate.

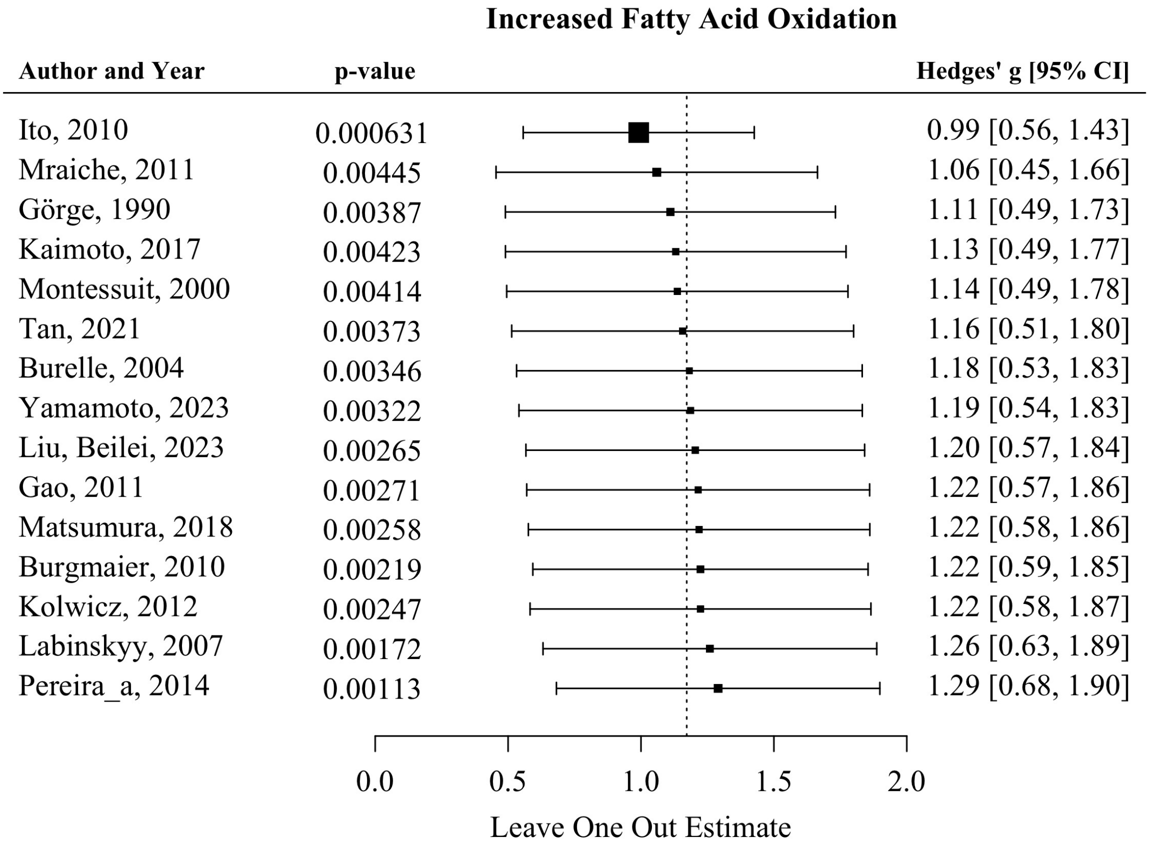

Supplementary Figure 4: Leave-one-out forest plot for interventions associated with increased cardiac fatty acid oxidation (FAO↑). For each study, the effect size displayed corresponds to an overall effect size calculated from a meta-analysis excluding that study. A vertical line at the overall effect size based on the complete set of studies (with no omission) is plotted to help detect influential studies. Data are presented as Hedges' g. Bars represent 95% CI. Tests for the overall effect and confidence intervals are based on the Knapp and Hartung method. Individual box sizes are determined by their weight in the meta-analysis model. Number of control (n = 127) and intervention group (n = 133) animals.

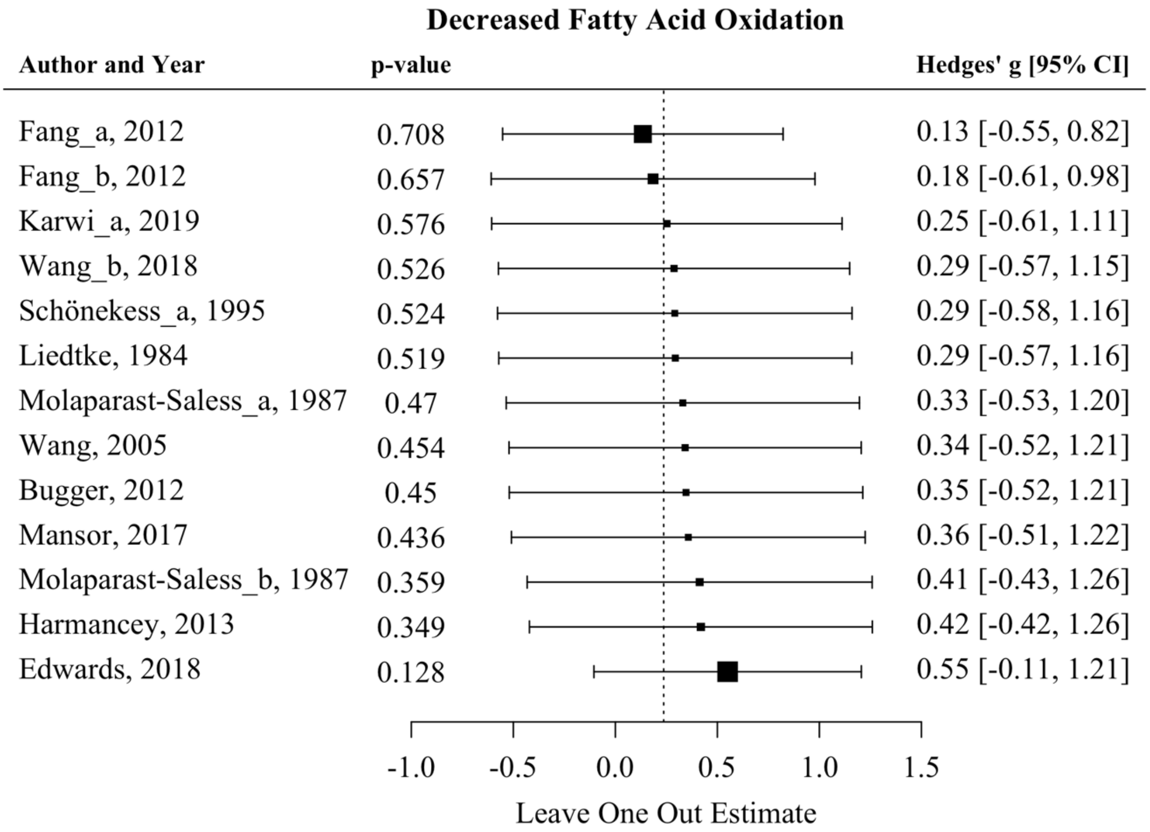

Supplementary Figure 5: Leave-one-out forest plot for interventions associated with decreased cardiac fatty acid oxidation (FAO↓). For each study, the effect size displayed corresponds to an overall effect size calculated from a meta-analysis excluding that study. A vertical line at the overall effect size based on the complete set of studies (with no omission) is plotted to help detect influential studies. Data are presented as Hedges' g. Bars represent 95% CI. Tests for the overall effect and confidence intervals are based on the Knapp and Hartung method. Individual box sizes are determined by their weight in the meta-analysis model. Number of control (n = 92) and intervention group (n = 88) animals.

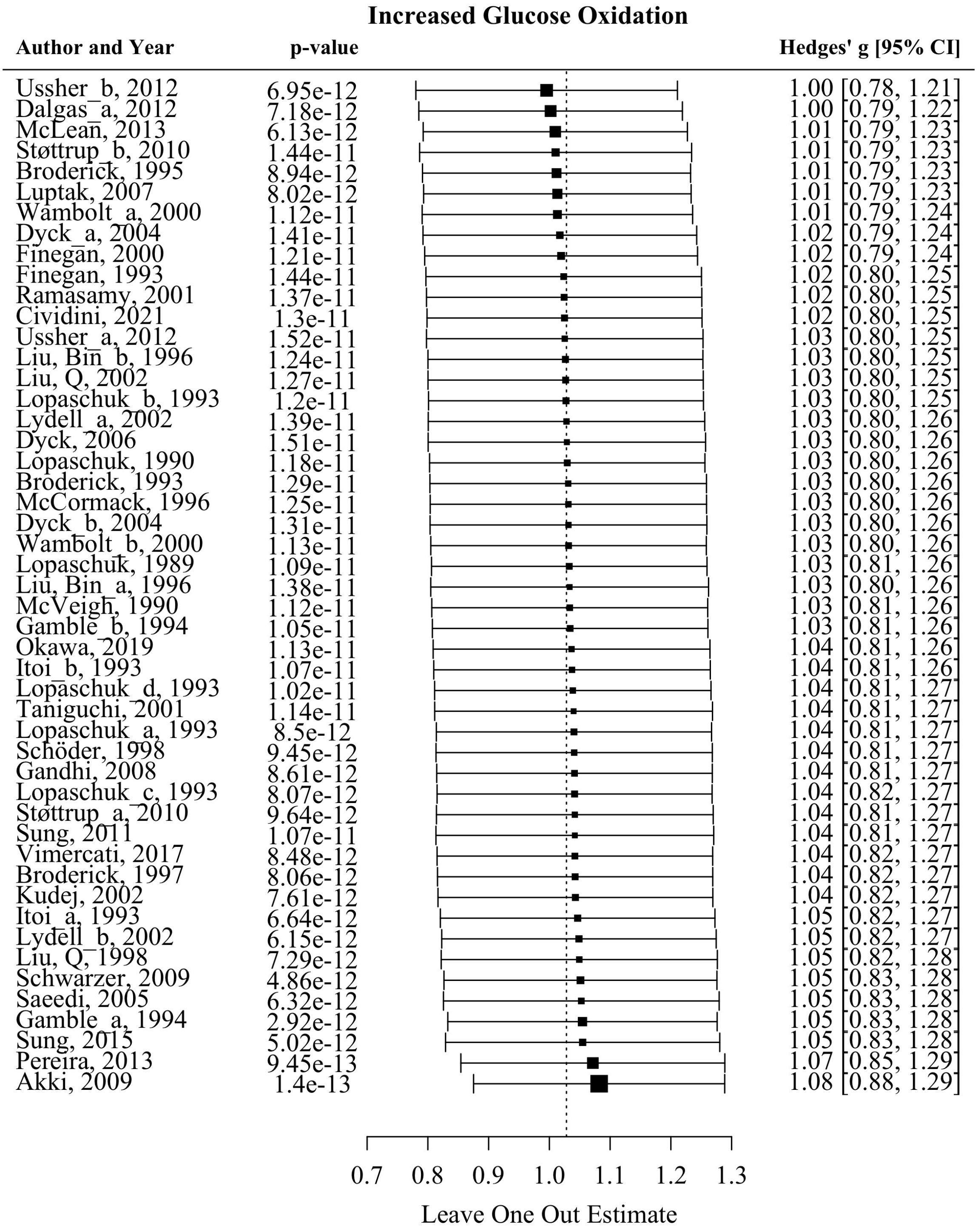

Supplementary Figure 6: Leave-one-out forest plot for interventions associated with increased cardiac glucose oxidation (GO↑). For each study, the effect size displayed corresponds to an overall effect size calculated from a meta-analysis excluding that study. A vertical line at the overall effect size based on the complete set of studies (with no omission) is plotted to help detect influential studies. Data

are presented as Hedges' g. Bars represent 95% CI. Tests for the overall effect and confidence intervals are based on the Knapp and Hartung method. Individual box sizes are determined by their weight in the meta-analysis model. Number of control (n = 393) and intervention group (n = 418) animals.

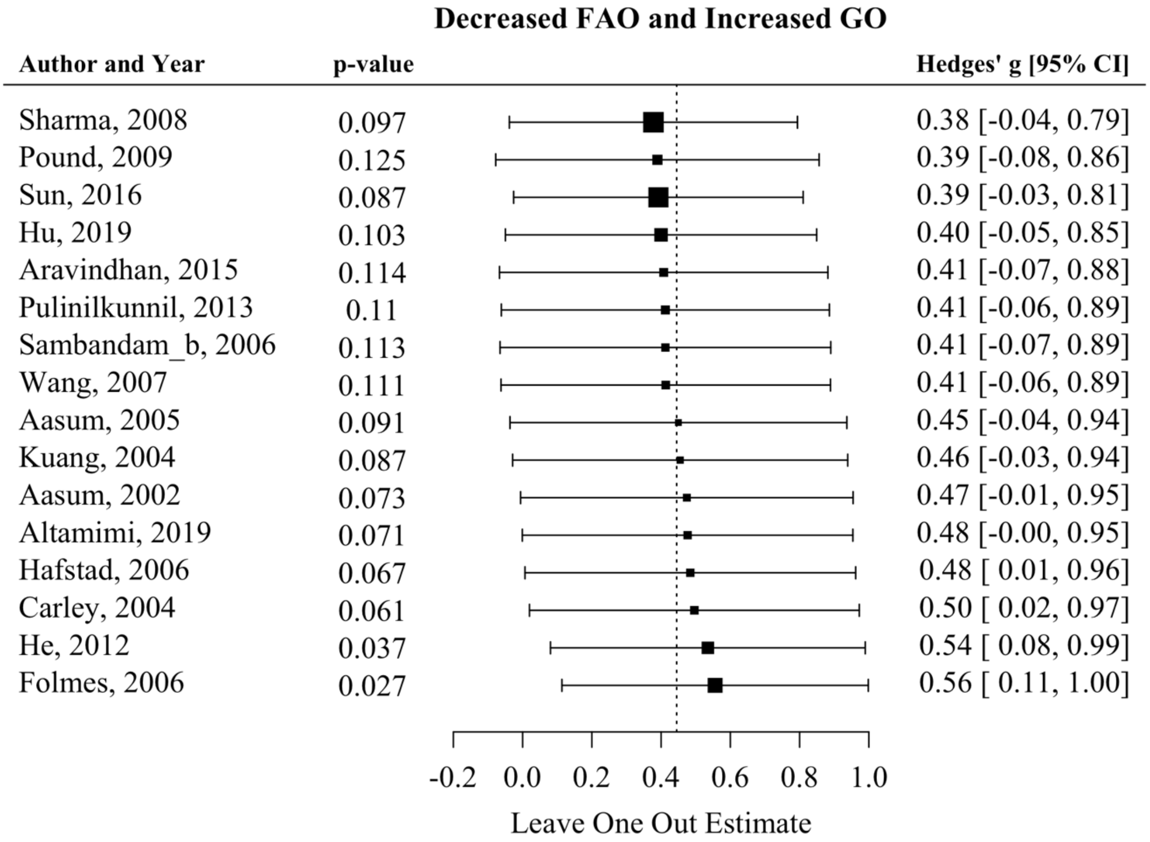

Supplementary Figure 7: Leave-one-out forest plot for interventions associated with decreased cardiac fatty acid oxidation and increased glucose oxidation (FAO↓GO↑). For each study, the effect size displayed corresponds to an overall effect size calculated from a meta-analysis excluding that study. A vertical line at the overall effect size based on the complete set of studies (with no omission) is plotted to help detect influential studies. Data are presented as Hedges' g. Bars represent 95% CI. Tests for the overall effect and confidence intervals are based on the Knapp and Hartung method. Individual box sizes are determined by their weight in the meta-analysis model. Number of control (n = 147) and intervention group (n = 147) animals.

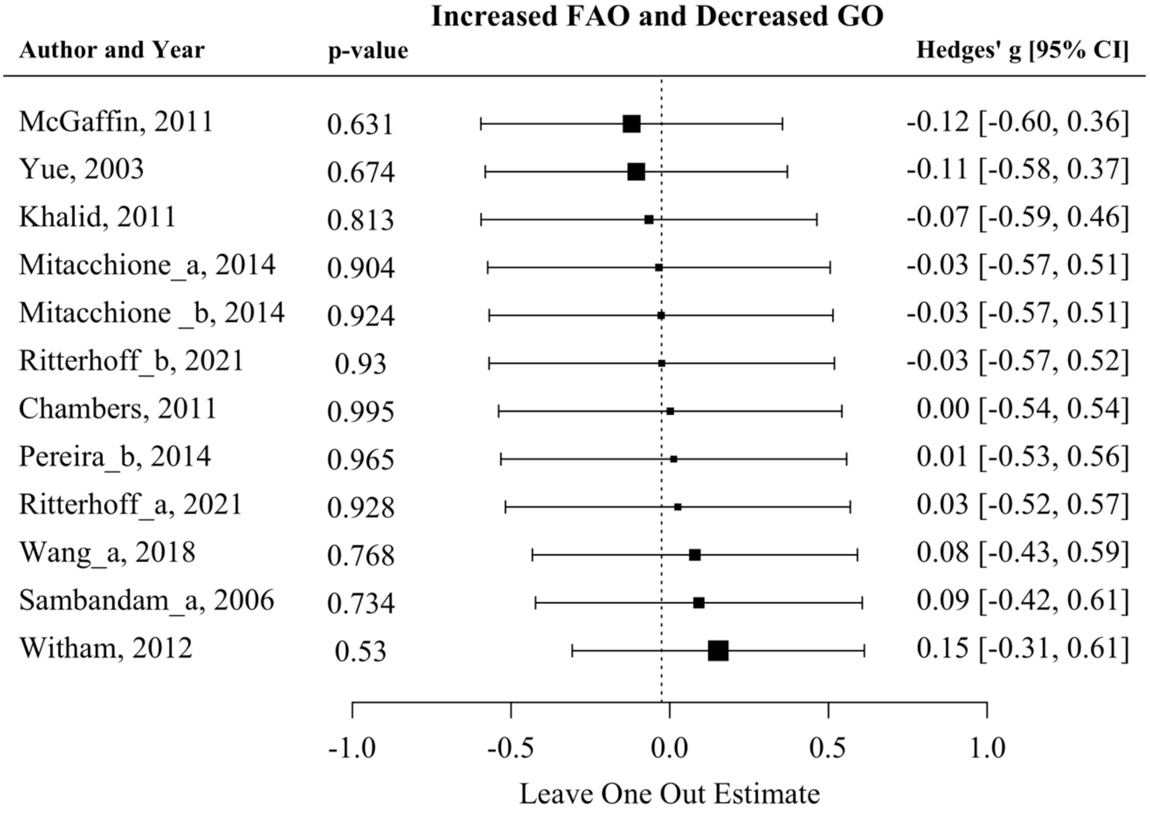

Supplementary Figure 8: Leave-one-out forest plot for interventions associated with increased cardiac fatty acid oxidation and decreased glucose oxidation (FAO↑GO↓). For each study, the effect size displayed corresponds to an overall effect size calculated from a meta-analysis excluding that study. A vertical line at the overall effect size based on the complete set of studies (with no omission) is plotted to help detect influential studies. Data are presented as Hedges' g. Bars represent 95% CI. Tests for the overall effect and confidence intervals are based on the Knapp and Hartung method. Individual box sizes are determined by their weight in the meta-analysis model. Number of control and (n = 96) intervention group (n = 100) animals.

Supplementary References

1 Liedtke, A. J., Nellis, S. H. & Mjos, O. D. Effects of reducing fatty acid metabolism on mechanical function in regionally ischemic hearts. *The American journal of physiology* **247**, H387-394 (1984). https://doi.org:doi:

2 Molaparast-Saless, F., Liedtke, A. J. & Nellis, S. H. Effects of the fatty acid blocking agents, oxfenicine and 4-bromocrotonic acid, on performance in aerobic and ischemic myocardium. *Journal of Molecular & Cellular Cardiology* **19**, 509-520 (1987). https://doi.org:doi:

3 Lopaschuk, G. D., McNeil, G. F. & McVeigh, J. J. GLUCOSE-OXIDATION IS STIMULATED IN REPERFUSED ISCHEMIC HEARTS WITH THE CARNITINE PALMITOYLTRANSFERASE-1 INHIBITOR, ETOMOXIR. *Molecular and Cellular Biochemistry* **88**, 175-179 (1989). https://doi.org:doi:

4 Gorge, G., Papageorgiou, I. & Lerch, R. Epinephrine-stimulated contractile and metabolic reserve in postischemic rat myocardium. *Basic research in cardiology* **85**, 595-605 (1990). https://doi.org:doi:

5 Lopaschuk, G. D., Spafford, M. A., Davies, N. J. & Wall, S. R. GLUCOSE AND PALMITATE OXIDATION IN ISOLATED WORKING RAT HEARTS REPERFUSED AFTER A PERIOD OF TRANSIENT GLOBAL-ISCHEMIA. *Circulation Research* **66**, 546-553 (1990). https://doi.org:doi:10.1161/01.Res.66.2.546

6 McVeigh, J. J. & Lopaschuk, G. D. DICHLOROACETATE STIMULATION OF GLUCOSE-OXIDATION IMPROVES RECOVERY OF ISCHEMIC RAT HEARTS. *American Journal of Physiology* **259**, H1079-H1085 (1990). https://doi.org:doi:10.1152/ajpheart.1990.259.4.H1079

7 Broderick, T. L., Quinney, H. A., Barker, C. C. & Lopaschuk, G. D. BENEFICIAL EFFECT OF CARNITINE ON MECHANICAL RECOVERY OF RAT HEARTS REPERFUSED AFTER A TRANSIENT PERIOD OF GLOBAL-ISCHEMIA IS ACCOMPANIED BY A STIMULATION OF GLUCOSE-OXIDATION. *Circulation* **87**, 972-981 (1993). https://doi.org:doi:10.1161/01.Cir.87.3.972

8 Finegan, B. A., Lopaschuk, G. D., Coulson, C. S. & Clanachan, A. S. Adenosine alters glucose use during ischemia and reperfusion in isolated rat hearts. *Circulation* **87**, 900-908 (1993). https://doi.org:doi:

9 Itoi, T., Huang, L. & Lopaschuk, G. D. GLUCOSE USE IN NEONATAL RABBIT HEARTS REPERFUSED AFTER GLOBAL-ISCHEMIA. *American Journal of Physiology* **265**, H427-H433 (1993). https://doi.org:doi:10.1152/ajpheart.1993.265.2.H427

10 Lopaschuk, G. D. *et al.* ISLET TRANSPLANTATION IMPROVES GLUCOSE-OXIDATION AND MECHANICAL FUNCTION IN DIABETIC RAT HEARTS. *Canadian Journal of Physiology and Pharmacology* **71**, 896-903 (1993). https://doi.org:doi:10.1139/y93-136

11 Lopaschuk, G. D., Wambolt, R. B. & Barr, R. L. AN IMBALANCE BETWEEN GLYCOLYSIS AND GLUCOSE-OXIDATION IS A POSSIBLE EXPLANATION FOR THE DETRIMENTAL EFFECTS OF HIGH-LEVELS OF FATTY-ACIDS DURING AEROBIC REPERFUSION OF ISCHEMIC HEARTS. *Journal of Pharmacology and Experimental Therapeutics* **264**, 135-144 (1993). https://doi.org:doi:

12 Gamble, J. & Lopaschuk, G. D. Glycolysis and glucose oxidation during reperfusion of ischemic hearts from diabetic rats. *Biochimica et biophysica acta* **1225**, 191-199 (1994). https://doi.org:doi:

13 Broderick, T. L., Quinney, H. A. & Lopaschuk, G. D. L-CARNITINE INCREASES GLUCOSE-METABOLISM AND MECHANICAL FUNCTION FOLLOWING ISCHEMIA IN DIABETIC RAT-HEART. *Cardiovascular Research* **29**, 373-378 (1995). https://doi.org:doi:10.1016/0008-6363(96)88594-4

14 Schonekess, B. O., Allard, M. F. & Lopaschuk, G. D. PROPIONYL L-CARNITINE IMPROVEMENT OF HYPERTROPHIED RAT-HEART FUNCTION IS ASSOCIATED

WITH AN INCREASE IN CARDIAC EFFICIENCY. *European Journal of Pharmacology* **286**, 155-166 (1995). https://doi.org:doi:10.1016/0014-2999(95)00442-n
15 Schonekess, B. O., Allard, M. F. & Lopaschuk, G. D. Propionyl L-carnitine improvement of hypertrophied heart function is accompanied by an increase in carbohydrate oxidation. *Circulation research* **77**, 726-734 (1995). https://doi.org:doi:
16 Broderick, T. L., Haloftis, G. & Paulson, D. J. L-propionylcarnitine enhancement of substrate oxidation and mitochondrial respiration in the diabetic rat heart. *Journal of Molecular & Cellular Cardiology* **28**, 331-340 (1996). https://doi.org:doi:
17 Itoi, T. & Lopaschuk, G. D. Calcium improves mechanical function and carbohydrate metabolism following ischemia in isolated Bi-ventricular working hearts from immature rabbits. *Journal of molecular and cellular cardiology* **28**, 1501-1514 (1996). https://doi.org:doi:
18 Liu, B., Clanachan, A. S., Schulz, R. & Lopaschuk, G. D. Cardiac efficiency is improved after ischemia by altering both the source and fate of protons. *Circulation research* **79**, 940-948 (1996). https://doi.org:doi:
19 Liu, B., el Alaoui-Talibi, Z., Clanachan, A. S., Schulz, R. & Lopaschuk, G. D. Uncoupling of contractile function from mitochondrial TCA cycle activity and MVO2 during reperfusion of ischemic hearts. *The American journal of physiology* **270**, H72-80 (1996). https://doi.org:doi:
20 McCormack, J. G., Barr, R. L., Wolff, A. A. & Lopaschuk, G. D. Ranolazine stimulates glucose oxidation in normoxic, ischemic, and reperfused ischemic rat hearts. *Circulation* **93**, 135-142 (1996). https://doi.org:doi:
21 Broderick, T. L., Currie, R. W. & Paulson, D. J. Heat stress induces vapid recovery of mechanical function of ischemic fatty acid perfused hearts by stimulating glucose oxidation during reperfusion. *Canadian Journal of Physiology and Pharmacology* **75**, 1273-1279 (1997). https://doi.org:doi:10.1139/y97-166
22 Liu, Q., Clanachan, A. S. & Lopaschuk, G. D. Acute effects of triiodothyronine on glucose and fatty acid metabolism during reperfusion of ischemic rat hearts. *The American journal of physiology* **275**, E392-399 (1998). https://doi.org:doi:https://dx.doi.org/10.1152/ajpendo.1998.275.3.E392
23 Schoder, H., Knight, R. J., Kofoed, K. F., Schelbert, H. R. & Buxton, D. B. Regulation of pyruvate dehydrogenase activity and glucose metabolism in post-ischaemic myocardium. *Biochimica et biophysica acta* **1406**, 62-72 (1998). https://doi.org:doi:
24 Broderick, T. L., Driedzic, W. & Paulson, D. J. Propionyl-L-carnitine effects on postischemic recovery of heart function and substrate oxidation in the diabetic rat. *Molecular and cellular biochemistry* **206**, 151-157 (2000). https://doi.org:doi:
25 Finegan, B. A., hi, M. & Clanachan, A. S. Phentolamine prevents the adverse effects of adenosine on glycolysis and mechanical function in isolated working rat hearts subjected to antecedent ischemia. *Journal of Molecular & Cellular Cardiology* **32**, 1075-1086 (2000). https://doi.org:doi:
26 Montessuit, C., Papageorgiou, I., Tardy-Cantalupi, I., Rosenblatt-Velin, N. & Lerch, R. Postischemic recovery of heart metabolism and function: role of mitochondrial fatty acid transfer. *Journal of Applied Physiology* **89**, 111-119 (2000). https://doi.org:doi:
27 Wambolt, R. B., Lopaschuk, G. D., Brownsey, R. W. & Allard, M. F. Dichloroacetate improves postischemic function of hypertrophied rat hearts. *Journal of the American College of Cardiology* **36**, 1378-1385 (2000). https://doi.org:doi:
28 Ramasamy, R., Hwang, Y., Bakr, S. & Bergmann, S. R. Protection of ischemic hearts perfused with an anion exchange inhibitor, DIDS, is associated with beneficial changes in substrate metabolism. *Cardiovascular Research* **51**, 275-282 (2001). https://doi.org:doi:
29 Taniguchi, M. *et al.* Dichloroacetate improves cardiac efficiency after ischemia independent of changes in mitochondrial proton leak. *American journal of physiology. Heart and circulatory physiology* **280**, H1762-1769 (2001). https://doi.org:doi:
30 Aasum, E. *et al.* Cardiac function and metabolism in type 2 diabetic mice after treatment with BM 17.0744, a novel PPAR-α activator. *American Journal of Physiology - Heart and Circulatory Physiology* **283**, H949-H957 (2002). https://doi.org:doi:10.1152/ajpheart.00226.2001

31 Kudej, R. K. *et al.* Brief increase in carbohydrate oxidation after reperfusion reverses myocardial stunning in conscious pigs. *Circulation* **106**, 2836-2841 (2002). https://doi.org:doi:

32 Liu, Q. *et al.* High levels of fatty acids delay the recovery of intracellular pH and cardiac efficiency in post-ischemic hearts by inhibiting glucose oxidation. *Journal of the American College of Cardiology* **39**, 718-725 (2002). https://doi.org:doi:

33 Lydell, C. P. *et al.* Pyruvate dehydrogenase and the regulation of glucose oxidation in hypertrophied rat hearts. *Cardiovascular research* **53**, 841-851 (2002). https://doi.org:doi:

34 Yue, T. L. *et al.* Activation of Peroxisome Proliferator-Activated Receptor-α Protects the Heart from Ischemia/Reperfusion Injury. *Circulation* **108**, 2393-2399 (2003). https://doi.org:doi:10.1161/01.CIR.0000093187.42015.6C

35 Burelle, Y. *et al.* Regular exercise is associated with a protective metabolic phenotype in the rat heart. *American journal of physiology. Heart and circulatory physiology* **287**, H1055-1063 (2004). https://doi.org:doi:

36 Carley, A. N. *et al.* Treatment of type 2 diabetic db/db mice with a novel PPAR-γ agonist improves cardiac metabolism but not contractile function. *American Journal of Physiology - Endocrinology and Metabolism* **286**, E449-E455 (2004). https://doi.org:doi:10.1152/ajpendo.00329.2003

37 Dyck, J. R. B. *et al.* Malonyl coenzyme a decarboxylase inhibition protects the ischemic heart by inhibiting fatty acid oxidation and stimulating glucose oxidation. *Circulation research* **94**, e78-84 (2004). https://doi.org:doi:

38 Kuang, M., Febbraio, M., Wagg, C., Lopaschuk, G. D. & Dyck, J. R. B. Fatty acid translocase/CD36 deficiency does not energetically or functionally compromise hearts before or after ischemia. *Circulation* **109**, 1550-1557 (2004). https://doi.org:doi:

39 Aasum, E., Cooper, M., Severson, D. L. & Larsen, T. S. Effect of BM 17.0744, a PPARalpha ligand, on the metabolism of perfused hearts from control and diabetic mice. *Canadian journal of physiology and pharmacology* **83**, 183-190 (2005). https://doi.org:doi:

40 Saeedi, R. *et al.* Trimetazidine normalizes postischemic function of hypertrophied rat hearts. *The Journal of pharmacology and experimental therapeutics* **314**, 446-454 (2005). https://doi.org:doi:

41 Wang, P., Lloyd, S. G. & Chatham, J. C. Impact of high glucose/high insulin and dichloroacetate treatment on carbohydrate oxidation and functional recovery after low-flow ischemia and reperfusion in the isolated perfused rat heart. *Circulation* **111**, 2066-2072 (2005). https://doi.org:doi:

42 Dyck, J. R. B. *et al.* Absence of malonyl coenzyme A decarboxylase in mice increases cardiac glucose oxidation and protects the heart from ischemic injury. *Circulation* **114**, 1721-1728 (2006). https://doi.org:doi:

43 Folmes, C. D. L., Clanachan, A. S. & Lopaschuk, G. D. Fatty acids attenuate insulin regulation of 5′-AMP-activated protein kinase and insulin cardioprotection after ischemia. *Circulation Research* **99**, 61-68 (2006). https://doi.org:doi:10.1161/01.RES.0000229656.05244.11

44 Hafstad, A. D., Solevag, G. H., Severson, D. L., Larsen, T. S. & Aasum, E. Perfused hearts from Type 2 diabetic (db/db) mice show metabolic responsiveness to insulin. *American journal of physiology. Heart and circulatory physiology* **290**, H1763-1769 (2006). https://doi.org:doi:

45 Samb *et al.* Chronic activation of PPARalpha is detrimental to cardiac recovery after ischemia. *American journal of physiology. Heart and circulatory physiology* **290**, H87-95 (2006). https://doi.org:doi:

46 Labinskyy, V. *et al.* Chronic activation of peroxisome proliferator-activated receptor-α with fenofibrate prevents alterations in cardiac metabolic phenotype without changing the onset of decompensation in pacing-induced heart failure. *Journal of Pharmacology and Experimental Therapeutics* **321**, 165-171 (2007). https://doi.org:doi:10.1124/jpet.106.116871

47 Luptak, I. *et al.* Long-term effects of increased glucose entry on mouse hearts during normal aging and ischemic stress. *Circulation* **116**, 901-909 (2007). https://doi.org:doi:

48 Wang, P. *et al.* A comparison between ranolazine and CVT-4325, a novel inhibitor of fatty acid oxidation, on cardiac metabolism and left ventricular function in rat isolated perfused heart during ischemia and reperfusion. *The Journal of pharmacology and experimental therapeutics* **321**, 213-220 (2007). https://doi.org:doi:

49 Broderick, T. L. ATP production and TCA activity are stimulated by propionyl-L-carnitine in the diabetic rat heart. *Drugs in R&D* **9**, 83-91 (2008). https://doi.org:doi:

50 Gandhi, M., Finegan, B. A., Clanachan, A. & S., e. Role of glucose metabolism in the recovery of postischemic LV mechanical function: effects of insulin and other metabolic modulators. *American journal of physiology. Heart and circulatory physiology* **294**, H2576-2586 (2008). https://doi.org:doi:https://dx.doi.org/10.1152/ajpheart.00942.2007

51 Sharma, V. *et al.* Metoprolol improves cardiac function and modulates cardiac metabolism in the streptozotocin-diabetic rat. *American journal of physiology. Heart and circulatory physiology* **294**, H1609-1620 (2008). https://doi.org:doi:https://dx.doi.org/10.1152/ajpheart.00949.2007

52 Akki, A. & Seymour, A. M. Western diet impairs metabolic remodelling and contractile efficiency in cardiac hypertrophy. *Cardiovascular Research* **81**, 610-617 (2009). https://doi.org:doi:

53 Folmes, C. D. L., Sowah, D., Clanachan, A., S., e. & Lopaschuk, G. D. High rates of residual fatty acid oxidation during mild ischemia decrease cardiac work and efficiency. *Journal of molecular and cellular cardiology* **47**, 142-148 (2009). https://doi.org:doi:https://dx.doi.org/10.1016/j.yjmcc.2009.03.005

54 Pound, K. M. *et al.* Substrate-enzyme competition attenuates upregulated anaplerotic flux through malic enzyme in hypertrophied rat heart and restores triacylglyceride content: attenuating upregulated anaplerosis in hypertrophy. *Circulation research* **104**, 805-812 (2009). https://doi.org:doi:https://dx.doi.org/10.1161/CIRCRESAHA.108.189951

55 Schwarzer, M. *et al.* The metabolic modulators, Etomoxir and NVP-LAB121, fail to reverse pressure overload induced heart failure in vivo. *Basic Research in Cardiology* **104**, 547-557 (2009). https://doi.org:10.1007/s00395-009-0015-5.

56 Burgmaier, M. *et al.* Metabolic adaptation follows contractile dysfunction in the heart of obese Zucker rats fed a high-fat "Western" diet. *Obesity* **18**, 1895-1901 (2010). https://doi.org:doi:

57 Ito, M. *et al.* High levels of fatty acids increase contractile function of neonatal rabbit hearts during reperfusion following ischemia. *American journal of physiology. Heart and circulatory physiology* **298**, H1426-1437 (2010). https://doi.org:doi:https://dx.doi.org/10.1152/ajpheart.00284.2009

58 Stottrup, N. B. *et al.* Inhibition of the malate-aspartate shuttle by pre-ischaemic aminooxyacetate loading of the heart induces cardioprotection. *Cardiovascular research* **88**, 257-266 (2010). https://doi.org:doi:https://dx.doi.org/10.1093/cvr/cvq205

59 Chambers, K. T. *et al.* Chronic inhibition of pyruvate dehydrogenase in heart triggers an adaptive metabolic response. *The Journal of biological chemistry* **286**, 11155-11162 (2011). https://doi.org:doi:https://dx.doi.org/10.1074/jbc.M110.217349

60 Gao, X.-M. *et al.* Deletion of macrophage migration inhibitory factor protects the heart from severe ischemia-reperfusion injury: a predominant role of anti-inflammation. *Journal of molecular and cellular cardiology* **50**, 991-999 (2011). https://doi.org:doi:https://dx.doi.org/10.1016/j.yjmcc.2010.12.022

61 Khalid, A. M. *et al.* Cardioprotective effect of the PPAR ligand tetradecylthioacetic acid in type 2 diabetic mice. *American Journal of Physiology - Heart & Circulatory Physiology* **300**, H2116-2122 (2011). https://doi.org:doi:

62 McGaffin, K., Witham, W., Yester, K. & O'Donnell, C. Leptin Modulates Cardiac Glucose Metabolism and Attenuates Injury in Acute Myocardial Infarction. *Circulation* **124** (2011). https://doi.org:doi:

63 Mraiche, F., Wagg, C. S., Lopaschuk, G. D. & Fliegel, L. Elevated levels of activated NHE1 protect the myocardium and improve metabolism following ischemia/reperfusion injury. *Journal of molecular and cellular cardiology* **50**, 157-164 (2011). https://doi.org:doi:https://dx.doi.org/10.1016/j.yjmcc.2010.10.016

64 Riehle, C. *et al.* PGC-1β deficiency accelerates the transition to heart failure in pressure overload hypertrophy. *Circulation Research* **109**, 783-793 (2011). https://doi.org:doi:10.1161/CIRCRESAHA.111.243964
65 Sung, M. *et al.* Improved cardiac metabolism and activation of the RISK pathway contributes to improved post-ischemic recovery in calorie restricted mice. *Journal of molecular medicine (Berlin, Germany)* **89**, 291-302 (2011). https://doi.org:doi:https://dx.doi.org/10.1007/s00109-010-0703-5
66 Bugger, H. *et al.* Genetic loss of insulin receptors worsens cardiac efficiency in diabetes. *Journal of molecular and cellular cardiology* **52**, 1019-1026 (2012). https://doi.org:doi:https://dx.doi.org/10.1016/j.yjmcc.2012.02.001
67 Dalgas, C., Povlsen, J. A., Lofgren, B., Erichsen, S. B. & Botker, H. E. Effects of fatty acids on cardioprotection by pre-ischaemic inhibition of the malate-aspartate shuttle. *Clinical and experimental pharmacology & physiology* **39**, 878-885 (2012). https://doi.org:doi:https://dx.doi.org/10.1111/j.1440-1681.2012.05749.x
68 Fang, Y.-H. *et al.* Therapeutic inhibition of fatty acid oxidation in right ventricular hypertrophy: exploiting Randle's cycle. *Journal of molecular medicine (Berlin, Germany)* **90**, 31-43 (2012). https://doi.org:doi:https://dx.doi.org/10.1007/s00109-011-0804-9
69 He, L. *et al.* Carnitine palmitoyltransferase-1b deficiency aggravates pressure overload-induced cardiac hypertrophy caused by lipotoxicity. *Circulation* **126**, 1705-1716 (2012). https://doi.org:doi:https://dx.doi.org/10.1161/CIRCULATIONAHA.111.075978
70 Kolwicz, S. C., Jr. *et al.* Cardiac-specific deletion of acetyl CoA carboxylase 2 prevents metabolic remodeling during pressure-overload hypertrophy. *Circulation research* **111**, 728-738 (2012). https://doi.org:doi:https://dx.doi.org/10.1161/CIRCRESAHA.112.268128
71 Ussher, J. R. *et al.* Stimulation of glucose oxidation protects against acute myocardial infarction and reperfusion injury. *Cardiovascular research* **94**, 359-369 (2012). https://doi.org:doi:https://dx.doi.org/10.1093/cvr/cvs129
72 Witham, W., Yester, K., O'Donnell, C. P. & McGaffin, K. R. Restoration of glucose metabolism in leptin-resistant mouse hearts after acute myocardial infarction through the activation of survival kinase pathways. *Journal of molecular and cellular cardiology* **53**, 91-100 (2012). https://doi.org:doi:https://dx.doi.org/10.1016/j.yjmcc.2012.03.016
73 Harmancey, R., Vasquez, H. G., Guthrie, P. H. & Taegtmeyer, H. Decreased long-chain fatty acid oxidation impairs postischemic recovery of the insulin-resistant rat heart. *FASEB journal : official publication of the Federation of American Societies for Experimental Biology* **27**, 3966-3978 (2013). https://doi.org:doi:https://dx.doi.org/10.1096/fj.13-234914
74 McLean, B. A. *et al.* Enhanced recovery from ischemia-reperfusion injury in PI3Kalpha dominant negative hearts: investigating the role of alternate PI3K isoforms, increased glucose oxidation and MAPK signaling. *Journal of molecular and cellular cardiology* **54**, 9-18 (2013). https://doi.org:doi:https://dx.doi.org/10.1016/j.yjmcc.2012.10.015
75 Pereira, R. O. *et al.* Inducible overexpression of GLUT1 prevents mitochondrial dysfunction and attenuates structural remodeling in pressure overload but does not prevent left ventricular dysfunction. *Journal of the American Heart Association* **2**, e000301 (2013). https://doi.org:doi:
76 Pulinilkunnil, T. *et al.* Myocardial adipose triglyceride lipase overexpression protects diabetic mice from the development of lipotoxic cardiomyopathy. *Diabetes* **62**, 1464-1477 (2013). https://doi.org:doi:
77 Witham, W. G., Yester, K. A. & McGaffin, K. R. A high leucine diet mitigates cardiac injury and improves survival after acute myocardial infarction. *Metabolism: clinical and experimental* **62**, 290-302 (2013). https://doi.org:doi:https://dx.doi.org/10.1016/j.metabol.2012.07.023
78 Mitacchione, G. *et al.* The gut hormone ghrelin partially reverses energy substrate metabolic alterations in the failing heart. *Circulation. Heart failure* **7**, 643-651 (2014). https://doi.org:doi:https://dx.doi.org/10.1161/CIRCHEARTFAILURE.114.001167
79 Pereira, R. O. *et al.* Maintaining PGC-1alpha expression following pressure overload-induced cardiac hypertrophy preserves angiogenesis but not contractile or mitochondrial function. *FASEB journal : official publication of the Federation of American Societies for*

Experimental Biology **28**, 3691-3702 (2014). https://doi.org:doi:https://dx.doi.org/10.1096/fj.14-253823
80 Pereira, R. O. *et al.* GLUT1 deficiency in cardiomyocytes does not accelerate the transition from compensated hypertrophy to heart failure. *Journal of molecular and cellular cardiology* **72**, 95-103 (2014). https://doi.org:doi:https://dx.doi.org/10.1016/j.yjmcc.2014.02.011
81 Vimercati, C. *et al.* Beneficial effects of acute inhibition of the oxidative pentose phosphate pathway in the failing heart. *American journal of physiology. Heart and circulatory physiology* **306**, H709-717 (2014). https://doi.org:doi:https://dx.doi.org/10.1152/ajpheart.00783.2013
82 Aravindhan, K. *et al.* Cardioprotection Resulting from Glucagon-Like Peptide-1 Administration Involves Shifting Metabolic Substrate Utilization to Increase Energy Efficiency in the Rat Heart. *PloS one* **10**, e0130894 (2015). https://doi.org:doi:https://dx.doi.org/10.1371/journal.pone.0130894
83 Sung, M. M. *et al.* Resveratrol treatment of mice with pressure-overloadinduced heart failure improves diastolic function and cardiac energy metabolism. *Circulation: Heart Failure* **8**, 128-137 (2015). https://doi.org:doi:10.1161/CIRCHEARTFAILURE.114.001677
84 Sun, W. *et al.* Cardiac-Specific Deletion of the Pdha1 Gene Sensitizes Heart to Toxicological Actions of Ischemic Stress. *Toxicological sciences : an official journal of the Society of Toxicology* **151**, 193-203 (2016). https://doi.org:doi:https://dx.doi.org/10.1093/toxsci/kfw035
85 Kaimoto, S. *et al.* Activation of PPAR-α in the early stage of heart failure maintained myocardial function and energetics in pressure-overload heart failure. *American Journal of Physiology - Heart and Circulatory Physiology* **312**, H305-H313 (2017). https://doi.org:doi:10.1152/ajpheart.00553.2016
86 Mansor, L. S. *et al.* Inhibition of sarcolemmal FAT/CD36 by sulfo-N-succinimidyl oleate rapidly corrects metabolism and restores function in the diabetic heart following hypoxia/reoxygenation. *Cardiovascular research* **113**, 737-748 (2017). https://doi.org:doi:https://dx.doi.org/10.1093/cvr/cvx045
87 Niederberger, P. *et al.* High pre-ischemic fatty acid levels decrease cardiac recovery in an isolated rat heart model of donation after circulatory death. *Metabolism: clinical and experimental* **71**, 107-117 (2017). https://doi.org:doi:https://dx.doi.org/10.1016/j.metabol.2017.03.007
88 Quan, N. *et al.* Sestrin2 prevents age-related intolerance to ischemia and reperfusion injury by modulating substrate metabolism. *FASEB journal : official publication of the Federation of American Societies for Experimental Biology* **31**, 4153-4167 (2017). https://doi.org:doi:https://dx.doi.org/10.1096/fj.201700063R
89 Edwards, K. S. *et al.* Uncoupling protein 3 deficiency impairs myocardial fatty acid oxidation and contractile recovery following ischemia/reperfusion. *Basic research in cardiology* **113**, 47 (2018). https://doi.org:doi:https://dx.doi.org/10.1007/s00395-018-0707-9
90 Matsumura, N. *et al.* Resveratrol improves cardiac function and exercise performance in MI-induced heart failure through the inhibition of cardiotoxic HETE metabolites. *Journal of molecular and cellular cardiology* **125**, 162-173 (2018). https://doi.org:doi:https://dx.doi.org/10.1016/j.yjmcc.2018.10.023
91 Wang, L. *et al.* Cardiomyocyte-specific deletion of Sirt1 gene sensitizes myocardium to ischaemia and reperfusion injury. *Cardiovascular research* **114**, 805-821 (2018). https://doi.org:doi:https://dx.doi.org/10.1093/cvr/cvy033
92 Altamimi, T. R. *et al.* A novel role of endothelial autophagy as a regulator of myocardial fatty acid oxidation. *The Journal of thoracic and cardiovascular surgery* **157**, 185-193 (2019). https://doi.org:doi:https://dx.doi.org/10.1016/j.jtcvs.2018.07.047
93 Hu, H. *et al.* The cardioprotective effects of carvedilol on ischemia and reperfusion injury by AMPK signaling pathway. *Biomedicine & pharmacotherapy = Biomedecine & pharmacotherapie* **117**, 109106 (2019). https://doi.org:doi:https://dx.doi.org/10.1016/j.biopha.2019.109106
94 Karwi, Q. G. *et al.* Weight loss enhances cardiac energy metabolism and function in heart failure associated with obesity. *Diabetes, obesity & metabolism* **21**, 1944-1955 (2019). https://doi.org:doi:https://dx.doi.org/10.1111/dom.13762

95 Karwi, Q. G. *et al.* Targeting the glucagon receptor improves cardiac function and enhances insulin sensitivity following a myocardial infarction. *Cardiovascular diabetology* **18**, 1 (2019). https://doi.org:doi:https://dx.doi.org/10.1186/s12933-019-0806-4
96 Okawa, Y. *et al.* Ablation of cardiac TIGAR preserves myocardial energetics and cardiac function in the pressure overload heart failure model. *American journal of physiology. Heart and circulatory physiology* **316**, H1366-H1377 (2019). https://doi.org:doi:https://dx.doi.org/10.1152/ajpheart.00395.2018
97 Quan, N. *et al.* Substrate metabolism regulated by Sestrin2-mTORC1 alleviates pressure overload-induced cardiac hypertrophy in aged heart. *Redox biology* **36**, 101637 (2020). https://doi.org:doi:https://dx.doi.org/10.1016/j.redox.2020.101637
98 Cividini, F. *et al.* Ncor2/PPARα-Dependent Upregulation of MCUb in the Type 2 Diabetic Heart Impacts Cardiac Metabolic Flexibility and Function. *Diabetes* **70**, 665-679 (2021). https://doi.org:doi:10.2337/DB20-0779
99 Li, X. *et al.* Direct Cardiac Actions of the Sodium Glucose Co-Transporter 2 Inhibitor Empagliflozin Improve Myocardial Oxidative Phosphorylation and Attenuate Pressure-Overload Heart Failure. *Journal of the American Heart Association* **10**, e018298 (2021). https://doi.org:doi:https://dx.doi.org/10.1161/JAHA.120.018298
100 Ritterhoff, J. *et al.* Increasing fatty acid oxidation elicits a sex-dependent response in failing mouse hearts. *Journal of molecular and cellular cardiology* **158**, 1-10 (2021). https://doi.org:doi:https://dx.doi.org/10.1016/j.yjmcc.2021.05.004
101 Tan, Y. *et al.* Short-term but not long-term high fat diet feeding protects against pressure overload-induced heart failure through activation of mitophagy. *Life sciences* **272**, 119242 (2021). https://doi.org:doi:https://dx.doi.org/10.1016/j.lfs.2021.119242
102 Liu, B. *et al.* Effect of SH2B1 on glucose metabolism during pressure overload-induced cardiac hypertrophy and cardiac dysfunction. *Clinical and experimental pharmacology & physiology* (2023). https://doi.org:doi:https://dx.doi.org/10.1111/1440-1681.13807
103 Yamamoto, T. *et al.* RIP140 deficiency enhances cardiac fuel metabolism and protects mice from heart failure. *The Journal of clinical investigation* **133** (2023). https://doi.org:doi:https://dx.doi.org/10.1172/JCI162309
