# Supplementary material for: Modulation of cardiac fatty acid or glucose oxidation to treat heart failure in preclinical models: a systematic review and meta-analysis
Source: Commun Med (Lond). 2025 Jun 4;5:213. doi: 10.1038/s43856-025-00924-5 (PMC12134058; doi:10.1038/s43856-025-00924-5)
Supplement: Supplementary file 2 — Description of Additional Supplementary Files [file 43856_2025_924_MOESM2_ESM.pdf]

Supplementary Data 1: Characteristics of all included studies.

DMC: diabetic cardiomyopathy, IR: ischemia-reperfusion, LVEF: left ventricular ejection fraction, LVFS: left ventricular fractional shortening, LVEDD: left ventricular end diastolic diameter, LVEDP: left ventricular end diastolic pressure, LVDevP: left ventricular developed pressure, Pacing: pacing-induced heart failure, PO: pressure overload, RPP: rate pressure product.

Supplementary Data 2: **CAMARADES checklist and assessment of attrition bias.**

CAMARADES checklist: (1) peer reviewed publication; (2) control of temperature; (3) allocation concealment; (4) random allocation to treatment or control; (5) blinded assessment of outcome; (6) sample size calculation; (7) compliance with animal welfare regulations; and (8) statement of potential conflict of interests.

Supplementary Data 3: **Numerical data of all included studies.**

This dataset provides the extracted numerical data from each study included in the analysis, along with study characteristics.

Supplementary Data 4: **Source data for Figure 6**

This dataset provides the numerical results underlying the individual points in Figure 6. FAO: fatty acid oxidation, GO: glucose oxidation, ↑ or ↓: increased or decreased.
